# Molecular Regulation of HIV-1 Expression and Persistence Across Diverse Cellular Reservoirs

**DOI:** 10.3390/ijms27073244

**Published:** 2026-04-02

**Authors:** Ashlin N. Álvarez-Flores, Fabiola I. Colón-Santiago, Naiara I. Hernández-Santisteban, Julieness M. Correa-Haifa, Samuel E. Caldero-Reyes, Glamaris N. Rosario-Sanfiorenzo, Giovanni O. Alicea-Pérez, Gabriela V. Arvelo-Colón, Amanda C. Rivera-Payán, Jeshua J. Colón-Fernández, Amanda S. Jové-Bravo, Carolina Nieves-Moreno, Génesis Matos-Morales, Yariselis Cardona-Maldonado, Agneris Z. Irizarry-Marquez, Solianne Martínez-Jiménez, Eduardo Álvarez-Rivera

**Affiliations:** 1 Department of Biology, Universidad de Puerto Rico, Arecibo, PR 00612, USA; ashlin.alvarez@upr.edu (A.N.Á.-F.); gabriela.arvelo@upr.edu (G.V.A.-C.); carolina.nieves3@upr.edu (C.N.-M.); 2 School of Medicine, Universidad Central del Caribe, Bayamón, PR 00960, USA; 122fcolon@uccaribe.edu; 3 Department of Science and Technology, Interamerican University Metropolitan Campus, San Juan, PR 00926, USA; naiarai.hernandezsan@intermetro.edu (N.I.H.-S.); glamaris.nicolerosar@intermetro.edu (G.N.R.-S.); amandac.riverapayan@intermetro.edu (A.C.R.-P.); jeshuaj.colon@intermetro.edu (J.J.C.-F.); amandas.jovebravo@intermetro.edu (A.S.J.-B.); genesis.matosmorales@intermetro.edu (G.M.-M.); agnerisz.irizarrymar@intermetro.edu (A.Z.I.-M.); 4 Department of Biology, Universidad de Puerto Rico, Bayamón, PR 00960, USA; julieness.correa@upr.edu (J.M.C.-H.); samuel.caldero@upr.edu (S.E.C.-R.); giovanni.alicea2@upr.edu (G.O.A.-P.); yariselis.cardona@upr.edu (Y.C.-M.); 5 Department of Neuroscience, School of Medicine, Universidad Central del Caribe, Bayamón, PR 00960, USA; solianne.martinez@uccaribe.edu; 6 Department of Microbiology and Immunology, School of Medicine, Universidad Central del Caribe, Bayamón, PR 00960, USA

**Keywords:** HIV-1 persistence, viral reservoirs, host restriction factors, transcriptional regulation, host–virus interactions, immune evasion, immune modulation, non-canonical reservoirs, cytoskeletal regulation, cellular microenvironment

## Abstract

Despite the remarkable success of antiretroviral therapy (ART) in suppressing human immunodeficiency virus type 1 (HIV-1) replication, viral persistence remains a major barrier to cure. This persistence is sustained by heterogeneous cellular reservoirs in which viral expression is tightly regulated by host-dependent molecular mechanisms. Beyond the canonical cluster of differentiation 4 (CD4+) T-cell reservoirs, HIV-1 establishes long-lived infection in myeloid cells, glial populations within the central nervous system (CNS), and additional non-canonical cellular niches, each characterized by distinct transcriptional, epigenetic, and immune environments. In this review, we synthesize recent advances in understanding how HIV-1 expression, latency, and reactivation are shaped across diverse susceptible cell types. We highlight cell-type-specific mechanisms governing viral integration, chromatin organization, transcriptional elongation, innate immune sensing, host restriction factors, and cytoskeletal regulation. Particular emphasis is placed on how host signaling pathways and immune microenvironments contribute to reservoir stability and heterogeneity, complicating eradication strategies. We further discuss immunomodulatory approaches that seek to modulate viral expression without exacerbating immune activation. By integrating molecular, cellular, and immunological perspectives, this review provides a framework for understanding HIV-1 persistence as a context-dependent process and underscores the need for cell-type-tailored strategies in HIV cure research.

## 1. Introduction

The human immunodeficiency virus type 1 (HIV-1) remains a chronic infection of global significance. Despite the effectiveness of antiretroviral therapy (ART) in suppressing viral replication and reducing morbidity, treatment does not eliminate integrated proviral DNA or the cells that harbor it. As a result, HIV-1 infection persists at both the individual and population levels. By the end of 2024, approximately 40 million individuals were living with HIV worldwide, with thousands of new diagnoses reported annually, even in regions with broad access to ART [1,2]. This persistent burden underscores that the principal barrier to a cure is not insufficient drug potency but rather the ability of the virus to persist in treated individuals. Notably, infected cells exhibit heterogeneous levels of viral activity, enabling immune evasion and long-term survival during therapy [3,4].

**Figure 1:**
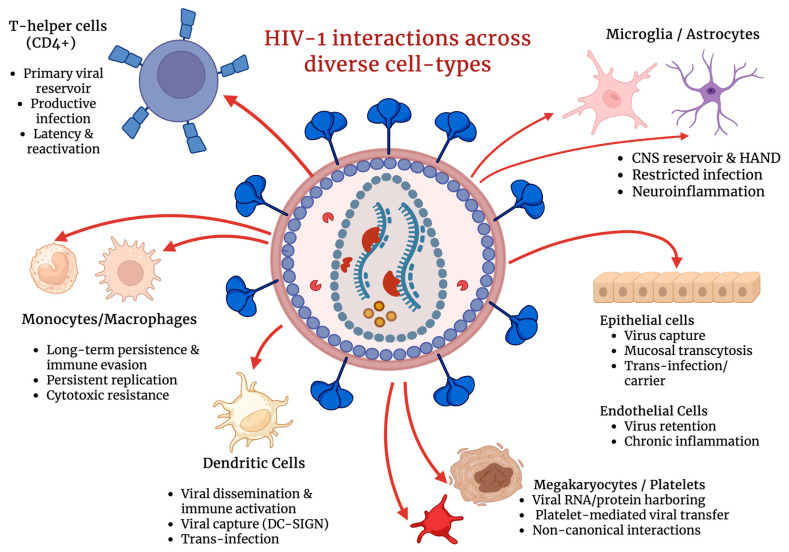
HIV-1 interactions and persistence across diverse cellular reservoirs. Schematic representation of the heterogeneous cellular landscape involved in HIV-1 infection, capture, and persistence. The central virion depicts the mature HIV-1 particle, while outward dark-red arrows indicate cell-type-specific interactions. Cluster of differentiation 4 (CD4) + T-helper (Th) cells represent the primary targets of productive infection and constitute the major latent viral reservoir capable of reactivation. Monocytes and macrophages support persistent viral replication and contribute to long-term viral survival by resisting cytopathic effects and immune evasion. Dendritic cells capture HIV-1 via lectin receptors such as DC-SIGN and promote viral dissemination and trans-infection of CD4+ T cells. Within the central nervous system, microglia and astrocytes exhibit restricted infection profiles and contribute to neuroinflammation and HIV-associated neurocognitive disorders (HAND). Epithelial cells facilitate viral capture and mucosal transcytosis and may act as carriers that enhance trans-infection of susceptible immune cells. Endothelial cells primarily mediate viral retention and endothelial activation associated with chronic inflammation rather than productive infection. Megakaryocytes and platelets can harbor viral RNA and proteins and participate in non-canonical platelet-mediated viral transfer. Collectively, the figure highlights the spectrum of productive, restricted, carriers, retention, and non-canonical HIV-1 interactions that contribute to viral dissemination, persistence, and immune dysregulation across anatomical compartments. Image created with BioRender.com.

The primary obstacle to HIV eradication is therefore not the lack of effective pharmacological suppression but the variability in viral expression across different cell types (Figure 1) and cellular states, which allows the virus to evade immune clearance and persist despite continuous ART [4]. Cluster of differentiation 4 (CD4+) T cells are particularly important in this context, as the clinically relevant viral reservoir is largely concentrated within long-lived memory subpopulations and quiescent cells, where transcriptional and epigenetic environments favor partial or intermittent viral silencing [5,6]. Recent studies emphasize the importance of examining rare infected CD4+ T cells under conditions that closely resemble their physiological state. Analyses that preserve cellular heterogeneity and avoid averaging effects across bulk populations have revealed associations between specific cellular phenotypes and distinct patterns of viral expression or latency that are otherwise obscured in population-level studies [5,7]. Although insights can be gained by examining viral activity across multiple susceptible cell types (Figure 1), understanding both the susceptibility and molecular mechanisms that regulate HIV expression within diverse CD4+ cells remains central to the development of effective cure strategies [4,5].

In this framework, transcription refers to the production of viral RNA from integrated proviral DNA and is strongly influenced by chromatin accessibility, epigenetic regulation, and the availability of host and viral transcriptional machinery. Factors such as chromatin compaction, transcription factor recruitment, and viral proteins, including Tat, play critical roles in enabling transcriptional elongation and driving the transition from a transcriptionally silent to an active state [6,8]. Latency describes a state in which viral expression is minimal or absent, allowing infected cells to evade immune-mediated clearance, whereas reactivation represents the exit from latency and is characterized by increased viral transcription in response to cellular activation or experimental perturbation [4,7]. Together, these processes define cellular reservoirs, populations of infected cells that sustain HIV-1 persistence and can drive rapid viral rebound upon treatment interruption [4].

Viral expression, latency, and reactivation arise from dynamic interactions among host signaling pathways, epigenetic and transcriptional regulation, cellular differentiation states, and immune pressures. An integrative framework that accounts for these host- and cell-specific factors is therefore essential for explaining reservoir heterogeneity and for developing mechanistically informed approaches to HIV cure research [4,5,9]. Within this context, immunomodulatory agents can serve as tools to investigate how controlled alterations in inflammatory tone or host signaling pathways influence cellular states associated with viral silencing, immune recognition, or persistence. Within this broader framework, host-directed immunomodulatory strategies have gained increasing attention as tools to influence HIV-1 expression without directly targeting viral enzymes. A range of natural compounds and biologically active agents have been shown to modulate innate and adaptive immune signaling pathways, inflammatory tone, and cellular activation states, thereby shaping cellular environments that may influence viral silencing, immune recognition, or persistence [10,11]. These approaches highlight the importance of examining HIV-1 expression through the lens of host immune regulation and cellular context, rather than exclusively through direct antiviral mechanisms.

In this comprehensive review, we highlight insights into the mechanisms governing HIV-1 expression, latency, and reactivation across diverse susceptible cell types (Figure 1), emphasizing how cell-specific regulatory networks contribute to viral persistence and immune evasion. Additionally, this review article aims to cover recent findings on how the highlighted immunomodulatory agents suppress or restrict viral expression and infectivity.

## 2. Key Transcriptional and Signaling Pathways in CD4+ T Cells

CD4+ T cells are essential helper lymphocytes that orchestrate and shape the immune responses through the secretion of soluble mediators and direct interactions with other immune cells [12]. The high expression of the CD4 receptor, as well as C-C chemokine receptor type 5 (CCR5) and C-X-C chemokine receptor type 4 (CXCR4) co-receptors, makes them the primary target for HIV infection (Figure 1), leading to acquired immunodeficiency syndrome (AIDS) upon long-lasting infection. Following entry, HIV integrates into the host genome of CD4+ T cells, establishing either ongoing infection or latency in resting memory cells.

One mechanism against HIV-1 involves transcriptional interference through the nuclear factor kappa B (NF-κB) pathway, which is essential for the virus’s transcription and plays a key role in latency reversal [13]. NF-κB functions as a master regulator of HIV transcription, which can lead to proviral expression. Activation of either canonical or noncanonical NF-κB pathways promotes recruitment of its subunits to the HIV long terminal repeats (LTR), thereby enhancing proviral transcription and replication. Conversely, a reduction in NF-κB signaling activity favors the establishment and maintenance of latency by limiting access to transcriptional machinery [14].

Beyond NF-κB, CD4+ T cells rely heavily on the Janus kinase (JAK)/signal transducer activator of transcription (STAT) signaling pathway. STAT is well-known to be a cytokine-driven transcriptional regulatory pathway that regulates the expression of CCR5, a critical co-receptor for R5-tropic HIV entry that influences infection susceptibility. Several categories of the STAT protein families, including STAT3, STAT5A, and STAT5B, upregulate CCR5, which results in an enhancement of susceptibility to R5-tropic HIV infection by CD4+ T cells [15]. STAT5 is one of the key proteins that enhances proviral transcription within CD4+ T cells by interacting with the LTR, supporting viral gene expression, leading to increased HIV virion production and viral spread. In contrast, STAT dysregulation caused by chronic HIV infection leads to immune exhaustion, contributing to long-lasting inflammation, impaired antiviral responses, and immunodeficiency [16].

**Figure 2:**
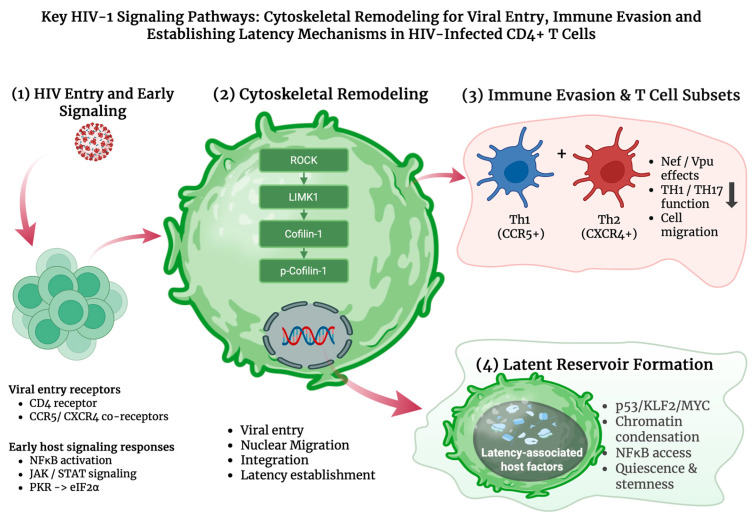
Key HIV-1 signaling pathways: cytoskeletal remodeling for viral entry, immune evasion, and establishment of latency in HIV-infected cluster of differentiation **4** (CD4+) T cells. Schematic overview of the coordinated host–virus interactions that facilitate HIV-1 entry, intracellular trafficking, immune modulation, and latent reservoir formation in CD4+ T cells. (1) HIV-1 entry is initiated through engagement of the CD4 receptor and CCR5/CXCR4 co-receptors. Receptor ligation can trigger early host signaling responses, including activation of nuclear factor kappa B (NFκB), the Janus kinase/signal transducer and activator of transcription (JAK/STAT) pathways, and protein kinase R (PKR)-mediated phosphorylation of eIF2α. (2) These events converge on cytoskeletal remodeling pathways regulated by the ROCK–LIMK1–cofilin axis, promoting actin dynamics that support CXCR4/CCR5 receptor clustering to CD4+, viral entry, nuclear migration, and proviral integration. (3) HIV-1 accessory proteins, including Nef and Vpu, contribute to immune evasion by impairing Th1/Th17 function and altering T-cell migration, with preferential involvement of CCR5+ Th1-like and CXCR4+ Th2-like subsets. (4) Following integration, infected CD4+ T cells may transition into a latent state characterized by chromatin condensation, restricted NFκB accessibility, and the induction of quiescent and stem-like phenotypes mediated in part by host transcriptional regulators such as p53, KLF2, and MYC. Colored nuclear icons in panel 4 represent these latency-associated host factors. Together, these interconnected mechanisms promote the establishment and persistence of latent HIV-1 reservoirs despite antiretroviral therapy (ART). Created with BioRender.com.

Because STAT pathways regulate interferon (IFN) responses, their dysregulation creates an antiviral imbalance that heightens cellular stress and increases reliance on other innate defenses, including the protein kinase R (PKR) pathway. PKR is activated by double-stranded RNA (dsRNA) and stress-related pathways, leading to the phosphorylation of eukaryotic translation initiation factor 2-alpha (eIF2α) (Figure 2) and inhibition of global protein synthesis through the unfolded protein response (UPR) as an antiviral defense mechanism [17]. While PKR has been shown to restrict HIV replication in vitro, the virus is able to counteract this response by regulating PKR activity or using accessory proteins such as negative regulatory factor (Nef) and Vpu to evade PKR-mediated restriction, highlighting a complex interplay between host defense and viral evasion [17]. Additionally, both Nef and Vpu are able to downregulate CD96 on CD4+ T cells, impairing CD96-mediated TH1/TH17 antiviral functions and cell migration, thereby revealing a previously unrecognized HIV-induced immune-evasion strategy (Figure 2) [18].

### 2.1. Cytoskeletal Regulation: Cofilin, LIMK1, and ROCK Signaling

During HIV infection, cofilin-1 activation in resting CD4+ T cells enhances actin dynamics, allowing the virus to overcome the cortical actin barrier that normally restricts entry and early post-entry steps. This cofilin-mediated remodeling, triggered by HIV envelope glycoprotein 120 (gp120) engagement with CXCR4 or CCR5, facilitates viral integration and promotes the establishment of latent infection in these cells [19]. CXCR4 triggers downstream signaling that can further promote cofilin activation and increase actin remodeling, a process shown to support efficient HIV entry into primary CD4+ T cells in CXCR4-tropic infections [20].

HIV-associated signaling reduces cofilin phosphorylation in resting CD4+ T cells, resulting in hyperactive cofilin that drives excessive actin treadmilling. This increased actin dynamics facilitates viral entry, nuclear migration, and integration while simultaneously disrupting normal cytoskeletal regulation and impairing CD4+ T-cell motility [21]. In resting memory CD4+ T cells, the chemokine interferon gamma-induced protein 10 (IP-10) enhances latent HIV infection by activating cofilin and actin remodeling. This effect could be blocked by inhibiting LIM kinase 1 (LIMK1), which prevents p-cofilin reduction and disrupts the actin rearrangements required for viral integration and reservoir establishment. Therefore, LIMK1 activity supports the actin cytoskeletal dynamics that enable HIV entry and its persistence (Figure 2) [20]. The Rho-associated protein kinase (ROCK) regulates LIMK1 and cofilin activity and supports HIV replication by promoting cytoskeletal remodeling and actomyosin contractility. These processes facilitate viral entry and intracellular trafficking in CD4+ T cells. Accordingly, inhibition of the ROCK–cofilin-1 pathway is known to reduce HIV infection, highlighting this signaling as a critical host factor exploited during the viral life cycle [22].

### 2.2. Establishing and Maintaining Latency in CD4+ T Cells

HIV establishes latency in quiescent memory CD4+ T cells by reprogramming them toward a resting state. Early activation of p53 and strong induction of Krüppel-like factor 2 (KLF2) downregulate myelocytomatosis oncogene (MYC) and other metabolic and transcriptional pathways, promoting a cellular environment that silences proviral transcription (Figure 2) [23]. Restrictive epigenetic structures further reinforce this silence by compacting chromatin around the provirus and limiting access to transcription factors such as NF-κB, thereby maintaining HIV in a deeply repressed state [24]. Latent infection persists in resting memory CD4+ cells, where integrated proviruses remain transcriptionally silent and produce non-detectable viral antigens, enabling them to evade immune clearance [25]. In addition, cluster of differentiation 8 (CD8+) cytotoxic T cells can further stabilize latency through noncytolytic mechanisms, driving metabolic and transcriptional quiescence in infected memory CD4+ T cells and promoting survival, stemness, and reduced activation. These are ideal conditions that suppress HIV transcription without eliminating infected cells. This non-cytolytic CD8-mediated suppression serves as an additional immune evasion mechanism that maintains latency until its removal allows viral reactivation [26].

HIV targets multiple T-helper (Th) lineages with varying efficiency. Th1 cells, which are known to express high levels of the CCR5 co-receptor, are among the earliest and most heavily infected subsets and also serve as major contributors to initial reservoir seeding. Infection is also detected in Th1/Th17 memory populations, while Th2 subsets, though less frequently infected early on, become strong viral targets as disease progresses due to their higher expression of the CXCR4 co-receptor (Figure 2) [27,28]. Across these lineages, HIV preferentially infects highly activated and proliferating cells, while long-lived memory subsets across Th1, Th2, and TH17 phenotypes enable persistent reservoir formation and continued viral survival (Figure 2). Notably, studies of HIV controllers demonstrate that potent, high-quality CD8+ T-cell responses play a critical role in viral inhibition. In particular, multifunctional cytotoxic CD8+ T cells and CXCR5-expressing subsets capable of accessing lymphoid tissues are associated with improved viral suppression, may contribute to enhanced immune surveillance, and may improve control of these HIV reservoirs [29]. These observations highlight the importance of effective cellular immunity in limiting persistent HIV infection. In contrast, regulatory and suppressive T-cell populations may facilitate evasion and long-term persistence.

### 2.3. Therapeutic Approaches Targeting HIV in CD4+ T Cells

A variety of therapeutic strategies for HIV have been developed to suppress HIV replication, limit disease progression, and potentially reduce the size of viral reservoirs within CD4+ T cells. Combination antiretroviral therapy (cART) remains the clinical standard and effectively suppresses viremia by inhibiting multiple steps of the viral life cycle. However, cART is not curative because treatment interruptions almost invariably lead to rapid viral rebound, driven by long-lived, latently infected CD4+ T cells that remain transcriptionally silent and insensitive to cART [30].

Beyond cART, immunomodulatory treatments, such as interleukin-7 (IL-7) and interleukin-15 (IL-15), have been studied for their ability to modulate proliferation in CD4+ and CD8+ T-cell populations, as well as natural killer (NK) cells, and influence HIV reservoir dynamics. IL-7 can expand both infected and uninfected CD4+ T cells and has been associated with latency reversal events that transiently increase HIV-1 RNA levels. IL-15 primarily expands CD8+ T cells and NK cells, potentially enhancing cytotoxic responses against infected cells and improving immune-mediated clearance [31].

A more recent study has suggested that long-acting injectable antiretroviral regimens offer an additional therapeutic avenue, providing durable suppression with infrequent dosing. Cabotegravir, an integrase strand transfer inhibitor, blocks the integration of viral DNA into the host genome, while rilpivirine, a non-nucleoside reverse transcriptase inhibitor, prevents reverse transcription. Together, these agents maintain virologic suppression of viral replication through infrequent intramuscular dosing and may improve treatment adherence in clinical settings [32].

## 3. Monocytes and Macrophages: A Natural Paradox in HIV

The mononuclear phagocyte system (MPS), composed of circulating monocytes and their tissue-differentiated descendants, macrophages, plays a paradoxical role in chronic viral infections (Figure 1). While these cells act as primary cellular defenders of the innate immune system, they can simultaneously contribute to HIV pathogenesis and prolonged viral persistence [33]. Monocytes originate in the bone marrow and circulate in the bloodstream before migrating into tissues, where they differentiate into monocyte-derived macrophages that mount an immune response, orchestrate tissue homeostasis, and participate in disease processes [34]. Although macrophages were historically believed to arise exclusively from circulating monocytes, it is now known that many tissue-resident macrophage populations, including microglia and Kupffer cells, originate from embryonic precursors. This developmental diversity creates a heterogeneous landscape with diverse functional properties across tissues [35,36].

Both monocytes and macrophages detect and eliminate pathogens, function as antigen-presenting cells (APCs), and participate in pro- and anti-inflammatory responses that contribute to tissue repair and immune regulation [34]. However, their functional versatility also allows these cells to establish cellular environments that support HIV infection and long-term persistence (Figure 1). Their longevity and resistance to cytopathic effects protect HIV from immune-mediated clearance and antiviral mechanisms [37]. Similar to CD4+ T cells, macrophages express the primary HIV entry receptor, CD4, and the co-receptors CCR5 and CXCR4, which enable viral envelope engagement and entry [38]. Consequently, the roles of monocytes and macrophages in viral persistence, innate immune signaling, and antigen presentation are among the central focuses of current HIV innate immunity studies.

### 3.1. Viral Persistence and Long-Term Reservoirs

Macrophages play a significant role in HIV persistence due to several intrinsic biological properties, including longevity, cellular heterogeneity, susceptibility to infection, self-renewal capacity, and resistance to cytopathic effects. These features enable macrophages to harbor latent viruses within tissues, such as the brain, gut, spleen, and urethra, despite individuals receiving ART [36]. Viral reservoirs are defined as cell populations that carry viral genomes capable of producing infectious virus following latency or treatment interruption. Macrophages are one of the populations that fit this criterion, as they are tissue-resident innate immune cells that support persistent HIV infection [35].

Additionally, macrophage polarization further contributes to their role as reservoirs for viruses. These highly plastic cells exhibit heterogeneity influenced by their interactions with different cytokines and tissue microenvironments [39]. Classically activated M1-type macrophages are typically associated with pro-inflammatory and anti-angiogenic responses, whereas alternatively activated M2-type macrophages promote tissue remodeling, angiogenesis, and immunoregulatory functions [40]. During chronic HIV infection, macrophages can transition toward intermediate or immunosuppressive polarization states, providing stable intracellular environments that are favorable for viral latency and reservoir maintenance [39].

In addition to polarization, macrophages can be categorized into two groups: tissue-resident or infiltrating populations. Tissue-resident macrophages, including microglia and alveolar macrophages, are maintained locally through self-renewal mechanisms rather than continuous monocytic replenishment [39]. Their persistence is regulated by signaling pathways involving the colony-stimulating factor 1 and its receptor (CSF-1/CSF-1R) and interleukin-34 (IL-34), which are known to support long-term survival and proliferation [41]. The prolonged lifespan and self-renewal capacity of these cells provide a stable niche in which HIV can persist in a latent state, largely protected from immune clearance and antiviral therapy.

Monocytes also play a critical role in the establishment, maintenance, and replenishment of HIV reservoirs. Originating from Ly6C^high^ precursors in the bone marrow, monocytes enter the circulation and tissues as a heterogeneous population that includes classical, intermediate, and non-classical subsets [42]. Although circulating monocytes have a relatively short lifespan, CD16+ monocyte subsets have been shown to contribute to viral persistence by sustaining HIV replication through cell-to-cell transmission and by infecting CD4+ T cells [43].

While there is a knowledge gap regarding which developmental stage these cells become infected, evidence further suggests that infection can occur at early stages of myeloid development, including in cluster of differentiation 34 (CD34+) hematopoietic stem cells and myeloid progenitors within the bone marrow [35]. During chronic infection, intermediate and non-classical monocytes exhibit increased susceptibility to HIV and can harbor proviral DNA even after long-term ART [34]. Latently infected circulating monocytes may infiltrate tissues and differentiate into macrophages, thereby seeding or replenishing tissue reservoirs. In contrast, uninfected circulating monocytes can migrate into tissues, become infected locally, and facilitate viral dissemination across anatomical compartments [35]. This cellular heterogeneity and migratory capacity to infiltrate tissue give monocytes the ability to replenish macrophage reservoirs and play a role in HIV persistence.

### 3.2. PKR, IFN Pathways, and Antiviral Restriction Mechanisms

Monocytes and macrophages are innate immune cells equipped with diverse pathogen-sensing receptors and signaling pathways, providing a first line of defense against viral infections through rapid, nonspecific responses [44]. Among the most important antiviral mechanisms in these cells are PKR and IFN signaling pathways, which, together, orchestrate cellular stress responses, antiviral gene expression, and potent immune regulation.

Viral infection is recognized in part through the detection of dsRNA, which activates PKR, a serine/threonine kinase involved in cellular stress response [45]. Upon activation, PKR phosphorylates eIF2α, inhibiting global mRNA translation and limiting viral protein synthesis [46]. While PKR activation can restrict viral replication and promote antiviral states, emerging evidence suggests that PKR signaling may also contribute to HIV persistence in a situational manner. Pharmacological modulation of PKR has been shown to impair HIV reverse transcription and integration in certain models, indicating that PKR activity can influence early stages of infection, autophagy, antigen presentation, and chronic inflammatory signaling, which collectively shape viral latency and reservoir maintenance [17].

In addition to blocking viral protein synthesis, PKR signaling is closely integrated with interferon-mediated responses in monocytes and macrophages. IFNs are a family of cytokines that can be classified into type I (e.g., IFN-α, IFN-β), type II (IFN-γ), and type III (IFN-λ) based on receptor usage [47]. During HIV infection, pathogen-associated molecular patterns (PAMPs) are detected by germline-encoded pattern-recognition receptors (PRRs), including toll-like receptors (TLRs), RIG-I receptors (RLRs), and NOD-like receptors (NLRs), which trigger innate immune signaling cascades that induce production of type I INFs in macrophages [47].

Binding of type I IFNs to the IFNα/β receptors (IFNAR), consisting of IFNAR1 and IFNAR2 subunits, activates the JAK/STAT signaling pathway, resulting in transcription of hundreds of interferon-stimulated genes (ISGs) that establish antiviral states [48]. In addition to canonical STAT-dependent signaling, IFN-α subtypes can activate non-canonical STAT-independent pathways that generate cell-type-specific ISG profiles, influencing HIV replication dynamics and reservoir environments [48]. Recent studies demonstrate that innate sensing of HIV RNA in macrophages can induce type I IFN responses that regulate proinflammatory cytokine production, limit viral spread, and shape the tissue reservoir environment, therefore amplifying antiviral defense [49].

Beyond cytokine signaling, monocytes and macrophages express intrinsic innate antiviral factors that directly inhibit HIV replication. One well-studied example is apolipoprotein B mRNA editing enzyme catalytic subunit 3G (APOBEC3G), an enzyme that introduces hypermutations into viral DNA during reverse transcription, disrupting viral replication [50]. However, this mechanism is counteracted by the viral protein Vif [50]. Other factors, such as bone marrow stromal antigen 2 (BST-2) (also known as Tetherin), an IFN-induced protein, prevent the release of nascent virions from infected cells, thereby reducing viral dissemination [51]. Sterile alpha motif and histidine–aspartate domain-containing protein 1 (SAMHD1), another critical restriction factor expressed in myeloid cells, restricts HIV replication by depleting intracellular deoxynucleoside triphosphate (dNTP) pools required for reverse transcription and can cooperate with IFN-induced MX2/MxB to block post-entry steps of viral replication [52]. Additional restriction mechanisms are actively being studied, including interferon-induced transmembrane (IFITM) family proteins and IFN-inducible E3 ligases, such as MARCH family members, which interfere with virion assembly, envelope incorporation, and cell-to-cell viral spread, further constraining HIV propagation [50].

### 3.3. Innate Immune Sensing and Viral Evasion in Monocytes and Macrophages

During HIV infection, innate immune sensing serves as a primary barrier for detecting and responding to viral presence in monocytes and macrophages. This process is often mediated by PRR, which recognizes PAMPs present in viral nucleic acids and proteins. Among these receptors, TLRs play a critical role in initiating innate immune responses by detecting viral components and triggering downstream signaling pathways that promote phagocytosis, cytokine production, and immune activation [53].

Upon engagement with viral PAMPs, PRRs activate intracellular signaling cascades that rely on transcription factors such as interferon regulatory factor 3 (IRF3) and NF-κB. These transcription factors drive the production of type I and type III IFNs, as well as pro-inflammatory cytokines, establishing antiviral states within infected and neighboring cells [54]. In addition to TLRs, cytosolic sensors, such as RIG-1-like receptors and melanoma differentiation-associated protein 5 (MDA5), detect dsRNA structures generated during viral replication. Activation of these receptors signals through the mitochondrial antiviral signaling protein (MAVS), leading to NFκB and IRF3 activation and subsequent IFN responses that restrict viral replication and shape inflammatory signaling in macrophages [55].

Despite robust innate immune sensing mechanisms, HIV has adapted and evolved multiple strategies to evade PRR-mediated detection and antiviral responses, thereby facilitating persistent infection. Viral accessory proteins such as Vif, Nef, and Vpr interfere with host restriction factors and early stages of the viral life cycle, while Vpu functions at a later stage to promote viral release and immune evasion [33]. At the same time, HIV exploits the migratory capacity of monocytes to disseminate to distant tissues, where infected or newly infected cells establish a latent reservoir and exhibit impaired antiviral responsiveness [56]. Overall, the interplay between innate immune sensing and viral evasion allows HIV to persist within monocytes and macrophages, reinforcing their role as long-term viral reservoirs.

### 3.4. Antigen Presentation and Immune Evasion in Monocytes and Macrophages

Beyond their role in innate sensing and viral restriction, monocytes and macrophages serve as a critical link between innate and adaptive immunity through their function as APCs. As professional APCs, macrophages internalize extracellular antigens, process them within the endocytic pathway, and load peptide fragments onto major histocompatibility complex class II (MHC-II) molecules within late endosomal compartments [57]. These peptide MHC-II complexes are subsequently transported to the cell surface, where they are recognized by T-cell receptors on CD4+ T-lymphocytes, enabling helper T-cell activation and adaptive immune coordination [57].

In parallel, APCs can present peptides derived from infected or ingested cells via major histocompatibility complex class I (MHC-I), leading to the activation of naive CD8+ T cells and their differentiation into cytotoxic T lymphocytes capable of eliminating antigen-expressing target cells [58]. Although dendritic cells are considered the most efficient and major APCs for T-cell priming, tissue-resident macrophages and monocyte-derived cells can also participate in cross-presentation, a process in which exogenous viral antigens are rerouted into the MHC-I pathway, thereby contributing to CD8+ T-cell activation [58].

Despite these immune-activating functions, HIV has evolved strategies to disrupt the antigen presentation process in macrophages and monocytes. One such example is the use of the viral accessory protein Nef, which interferes with transcription, trafficking, and surface expression of MHC class I and II molecules, thereby impairing antigen presentation and reducing effective activation of CD4+ and CD8+ T cells [59]. This disruption of the adaptive immune system represents a key immune evasion strategy that facilitates viral persistence and reservoir maintenance in myeloid cells.

## 4. Microglia and Astrocytes During HIV Infection in the Central Nervous System

The central nervous system (CNS) is a unique immunological compartment in HIV-1 infection. Unlike peripheral tissues, the CNS is characterized by tightly regulated immune surveillance, immune privilege, and limited regenerative capacity. Protective structures such as the blood–brain barrier (BBB) restrict immune cell entry and significantly limit the penetration of many antiretroviral drugs, creating an environment in which viral clearance is inherently challenging [4]. The maintenance of CNS immune homeostasis is largely mediated by resident glial cells, particularly microglia and astrocytes, which play central roles in immune regulation and neuronal support [60,61]. Despite these protective mechanisms, HIV-1 invades the CNS early following systemic infection.

Early seeding of the CNS enables HIV-1 to persist within long-lived cellular populations even when viral replication is effectively suppressed in peripheral tissues [62]. Consequently, the CNS can function as a semi-independent viral reservoir in which HIV persistence is partially uncoupled from systemic infection levels. This compartmentalization provides a mechanistic explanation for the continued presence of neurological complications in individuals with otherwise well-controlled systemic HIV infection. Indeed, viral persistence within the CNS is closely associated with the development of HIV-associated neurocognitive disorders (HAND), which continue to affect a substantial proportion of people living with HIV despite sustained plasma viral suppression [61].

**Figure 3:**
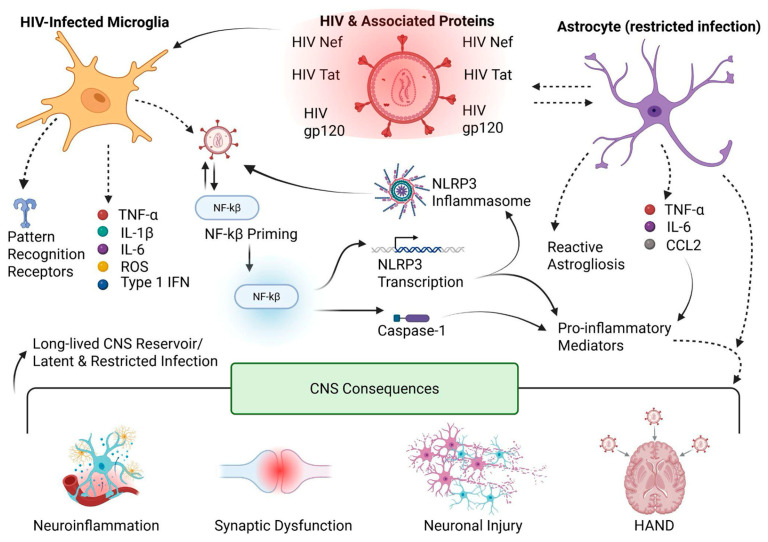
Interplay between HIV-infected microglia and astrocytes in CNS inflammation and neuropathogenesis. Microglia represent the primary HIV-1–infected myeloid cell population in the central nervous system and can function as long-lived viral reservoirs exhibiting latent or restricted infection. Viral components and HIV-associated proteins, including transactivator of transcription (Tat), Nef, and glycoprotein (gp120), together with pathogen-recognition receptor (PRR) signaling, activate nuclear factor kappa B (NF-κB)-dependent priming pathways that promote transcription and assembly of the NOD-like receptor family pyrin domain-containing 3 (NLRP3) inflammasome in microglia. Inflammasome activation leads to caspase-1 activation and the production of pro-inflammatory mediators, including tumor necrosis factor alpha (TNF-α), interleukin-1 beta (IL-1β), interleukin-6 (IL-6), reactive oxygen species (ROS), and type I interferons (IFN), which contribute to chronic neuroinflammation. Astrocytes, which typically support restricted or non-productive HIV infection, respond to both viral proteins and inflammatory mediators released from infected microglia. Exposure to Tat, Nef, gp120, and microglia-derived cytokines promotes reactive astrogliosis and the release of additional inflammatory factors, including TNF-α, IL-6, and C-C motif chemokine ligand 2 (CCL2), further amplifying the neuroinflammatory environment. This bidirectional signaling between microglia and astrocytes sustains chronic central nervous system (CNS) inflammation and contributes to neuronal dysfunction. Persistent glial activation and inflammatory signaling ultimately drive neuroinflammation, synaptic dysfunction, neuronal injury, and the development of HIV-associated neurocognitive disorders (HAND) despite suppressive antiretroviral therapy. Image created with BioRender.com.

HAND encompasses a spectrum of neurocognitive impairments that cannot be attributed to alternative neurological conditions and includes asymptomatic neurocognitive impairment, mild neurocognitive disorder, and HIV-associated dementia [61,63]. Current evidence indicates that HAND arises primarily from chronic immune dysregulation rather than direct neuronal infection [64]. Neurons are rarely productively infected by HIV-1; instead, progressive neurological injury results from prolonged exposure to inflammatory mediators, sustained glial activation, and disruption of neuronal support functions. Additional contributing factors may include long-term antiretroviral neurotoxicity [63]. In this context, glial cells, in particular microglia and astrocytes, emerge as central regulators of HIV persistence, immune modulation, and CNS injury (Figure 1 and Figure 3). Their innate immune responsiveness, intimate interactions with neurons, and extended lifespan position them as key drivers of HIV-associated neuropathogenesis [60,65].

### 4.1. Microglia: Latency, Restricted Replication, and CNS Reservoir Formation

Microglia are the resident macrophages of the CNS and constitute the primary immune-responsive cell population within the brain parenchyma. Under physiological conditions, microglia continuously survey the CNS environment, remove cellular debris, and regulate synaptic remodeling [60,66]. During HIV infection, these same immune functions, together with their long lifespan, position microglia as central contributors to viral persistence within the brain [67,68]. Although microglia express relatively low levels of CD4 and chemokine co-receptors compared with classical HIV target cells, such as CD4+ T lymphocytes, HIV-1 can nevertheless infect microglia through both conventional and alternative entry mechanisms [69,70].

Viral entry into microglia is generally less efficient than in peripheral immune cells, and intrinsic antiviral defenses are more actively engaged. Limited receptor availability, together with the presence of myeloid-specific restriction factors, results in a pattern of restricted viral replication that clearly distinguishes microglial infection from productive infection in activated CD4+ T cells [61,71]. In many cases, microglial infection is characterized by low-level viral gene expression rather than robust virion production, reflecting a latency-prone infection phenotype.

Following viral entry into the microglial plasma membrane, HIV-1 establishes infection primarily through CCR5-mediated fusion, although alternative mechanisms, such as cell-to-cell transfer from infected monocytes or macrophages that have crossed the BBB, have also been described [61,69]. After membrane fusion, the viral capsid is released into the cytoplasm, where reverse transcription occurs, followed by nuclear import of the pre-integration complex. HIV-1 then integrates into the host microglial genome and may subsequently transition into a transcriptionally silent or low-expression state, thereby establishing proviral latency [71,72].

In this latent state, viral gene expression is markedly suppressed, allowing HIV-1 to evade immune surveillance and antiretroviral drugs that primarily target active replication. Mechanistically, microglial latency is supported by epigenetic repression at the HIV LTR, including chromatin compaction and histone deacetylation that reduce promoter accessibility and limit transcriptional machinery recruitment [72]. In parallel, the CNS microenvironment can limit the availability or activation of key transcriptional drivers needed for robust HIV transcription, particularly NF-κB and nuclear factor of activated T cells (NFAT), making proviral activation less efficient than in activated peripheral CD4+ T cells [61,72]. Post-entry restriction further contributes to viral silencing, as SAMHD1, a restriction factor previously discussed in the context of myeloid antiviral defense (Section 3), reduces intracellular dNTP pools, thereby limiting reverse transcription efficiency and favoring low-expression or transcriptionally silent infection phenotypes in myeloid-lineage cells, including microglia [71]. Finally, CNS cellular context matters, as neuron-to-microglia cross-talk can suppress microglial activation programs and stabilize proviral quiescence, decreasing the likelihood of inflammation-driven reactivation and reinforcing long-term persistence within the brain [69,72].

Beyond transcriptional silencing, HIV-1 infection also reshapes microglial innate immune signaling through inflammasome activation, providing a direct mechanistic link between microglial infection and chronic CNS inflammation (Figure 1 and Figure 3). Inflammasome pathways (commonly discussed in the context of NLRP3 signaling) can promote caspase-1 activation and the maturation and release of interleukin-1 beta (IL-1β) and IL-18, which sustain neuroinflammation and propagate inflammatory signaling to surrounding glia and neurons. Importantly, this inflammatory state can persist even when productive viral replication is restricted, supporting a model in which HIV persistence and immune activation are partially uncoupled within the CNS (Figure 3) [64,73]. Whether sustained neuroinflammation in HAND requires ongoing low-level HIV replication within microglia remains an area of active investigation. Although HIV DNA and transcriptional activity have been detected in CNS-resident myeloid cells in vivo, evidence for persistent productive replication during suppressive ART is limited and appears to vary across patients and disease stages. These observations suggest that both residual viral activity and replication-independent inflammatory mechanisms may contribute to HAND pathogenesis [67].

Viral proteins can further amplify these pathways even when replication is limited. In particular, Nef has been linked to enhanced inflammatory signaling and altered intercellular communication in CNS myeloid cells, which may indirectly influence latency stability by maintaining a primed inflammatory state capable of episodic proviral reactivation. Consistent with this, infected human microglia exhibit activation of inflammatory programs associated with HAND pathogenesis [64,74].

Collectively, these mechanisms permit HIV-1 persistence within microglia while minimizing cytopathic effects and immune detection [68]. Because microglia are long-lived and capable of local self-renewal, latently infected microglia represent a particularly stable and clinically relevant viral reservoir within the CNS. This reservoir poses a major challenge for HIV cure strategies, as viral reactivation within the brain carries the risk of exacerbating neuroinflammation and neuronal injury [4,69].

### 4.2. Astrocyte Identity and Entry Mechanisms

Astrocytes constitute the most abundant glial cell population in the CNS and play essential roles in maintaining neuronal health. They regulate neurotransmitter clearance, metabolic support, ion homeostasis, synaptic stability, and BBB integrity [65,75]. Although astrocytes are not classical immune cells, they are highly responsive to inflammatory signals and play a significant role in CNS immune regulation [60]. In contrast to microglia, astrocytes are largely CD4-negative and do not support classical HIV entry pathways. Instead, HIV-1 can interact with astrocytes through CD4-independent mechanisms, including endocytosis and direct cell-to-cell transfer.

As a result, astrocytic infection is typically non-productive or highly restricted, with minimal viral replication and limited viral gene expression [75]. Despite this restricted infection profile, astrocytes can harbor integrated proviral DNA and express low levels of viral transcripts, fulfilling key criteria for viral persistence within the CNS [61]. Although astrocytes do not produce significant quantities of the infectious virus, infection can nevertheless disrupt their normal supportive functions.

Astrocytic persistence is further supported by latency-like blocks that occur at multiple stages of the viral life cycle. Because astrocytes lack classical CD4-mediated entry pathways, infection is frequently initiated through CD4-independent uptake routes, such as endocytosis or cell-to-cell transfer, which can result in inefficient fusion, uncoating, and incomplete replication programs [61,75]. Even when proviral DNA becomes detectable, astrocytes often exhibit restricted transcriptional and post-transcriptional activity, leading to low viral RNA output and minimal infectious particle production (Figure 1 and Figure 3). This phenotype is consistent with a cellular environment in which proviral expression is constrained by transcription factor availability and epigenetic regulation, permitting long-term viral persistence with minimal cytopathic effect while still enabling inflammatory modulation of the CNS microenvironment [61,75].

HIV-associated alterations in astrocytic metabolism, neurotransmitter regulation, and BBB maintenance increase neuronal vulnerability and promote CNS dysfunction. These effects are largely indirect and arise from astrocyte-mediated disturbances in neuronal support rather than from direct viral cytotoxicity. Over time, such disruptions exacerbate neuronal stress, impair synaptic function, and weaken the CNS microenvironment, thereby contributing to the cognitive impairment observed in HAND [65,75]. Importantly, astrocytosis in individuals living with HIV can be triggered not only by direct viral infection but also by exposure to viral proteins and inflammatory mediators released from infected macrophages and microglia. This reactive astrocyte phenotype is strongly influenced by HIV-derived proteins, among which Tat, Nef, and gp120 play particularly prominent roles (Figure 3) [76,77].

The HIV-1 transactivator of transcription (Tat) protein is actively released from infected cells and taken up by astrocytes, even in the absence of productive infection. Within astrocytes, Tat disrupts calcium homeostasis and mitochondrial function and impairs glutamate uptake by downregulating excitatory amino acid transporters. These alterations increase extracellular glutamate concentrations, promoting excitotoxic neuronal injury. Tat also activates pro-inflammatory signaling pathways, including NF-κB and STAT3, leading to increased cytokine and chemokine production that amplifies neuroinflammation. Through these mechanisms, Tat shifts astrocytes from a neuroprotective phenotype toward a pro-inflammatory state that contributes to sustained neuronal stress and CNS injury [65,76].

In addition to Tat, the HIV-1 Nef contributes to astrocyte-mediated neuropathogenesis. Although astrocytes do not support efficient viral replication, Nef can be expressed intracellularly or transferred between cells via extracellular vesicles. Within astrocytes, Nef disrupts cytoskeletal organization and vesicular trafficking, thereby altering pathways involved in BBB support and intercellular communication. These alterations are associated with dysregulated cytokine release and enhanced inflammatory signaling, as well as compromised astrocyte-endothelial interactions that facilitate immune cell infiltration into the CNS. Collectively, Nef-mediated astrocyte dysfunction intensifies neuroinflammation and indirectly contributes to neuronal impairment and cognitive decline [70,74].

The HIV-1 gp120 also plays a critical role in astrocyte-associated CNS injury. gp120 interacts with chemokine receptors such as CCR5 and CXCR4 expressed on astrocytes, triggering intracellular signaling cascades that promote astrocyte activation and inflammatory gene expression. Exposure to gp120 induces the release of pro-inflammatory cytokines, nitric oxide, and reactive oxygen species, leading to oxidative stress and synaptic dysfunction. In addition, gp120 disrupts astrocytic regulation of glutamate transporters, further enhancing excitotoxic neuronal damage. Importantly, these effects occur independently of active viral replication, illustrating how viral protein exposure alone can drive progressive neuronal injury and chronic neuroinflammation during HIV infection [73]. Nevertheless, the pathological impact of soluble gp120 in vivo is likely influenced by its effective tissue concentration, persistence, and local viral activity, as extracellular gp120 may be subject to rapid clearance and neutralization. It may also require sustained viral production to reach biologically significant levels [77].

### 4.3. Neuroinflammatory Microenvironment and Immune Modulation

HIV infection of microglia and astrocytes substantially alters the CNS immune landscape. Upon activation, HIV-infected microglia release a broad range of pro-inflammatory mediators, including tumor necrosis factor alpha (TNF-α), IL-1β, interleukin-6 (IL-6), and type I IFN. While these cytokines are essential components of antiviral defense, prolonged or dysregulated production of these cytokines promotes chronic neuroinflammation, synaptic injury, and neuronal stress [64]. Over time, this inflammatory environment disrupts neuronal communication and contributes to the cognitive and behavioral impairments characteristic of HAND [62,64].

Importantly, inflammatory signaling within the CNS may also promote episodic reactivation of latent provirus in microglia, thereby reinforcing cycles of immune activation and viral persistence.

Astrocytes further shape this inflammatory milieu by releasing cytokines and chemokines that influence both microglial activation and neuronal function. Disruption of astrocytic regulation of neurotransmitter clearance, metabolic coupling, and BBB integrity contributes to a sustained pro-inflammatory state that persists even in individuals receiving effective ART [65]. Continuous bidirectional communication between microglia and astrocytes thus plays a central role in maintaining chronic CNS inflammation during HIV infection (Figure 3).

### 4.4. Therapeutic Implications of Glial HIV Persistence

From a therapeutic perspective, HIV persistence within microglia and astrocytes presents substantial challenges for both viral suppression and cure strategies. Although ART effectively controls systemic viral replication, it fails to eradicate latent reservoirs within the CNS. Limited penetration of many antiretroviral drugs across the BBB, together with the transcriptionally silent nature of proviral genomes in glial cells, reduces therapeutic efficacy within this compartment [62]. Importantly, suboptimal antiretroviral drug penetration is not restricted to the CNS. Heterogeneous tissue exposure has also been documented in peripheral anatomical compartments, including lymph nodes, gut-associated lymphoid tissue, and other tissue reservoirs. Incomplete drug distribution within these sites may permit ongoing viral persistence despite otherwise suppressive systemic ART, highlighting tissue pharmacology as a critical determinant of reservoir stability [78].

Strategies aimed at reversing latency must therefore be approached with caution, as viral reactivation in the CNS carries the risk of exacerbating neuroinflammation and neuronal injury. Likewise, immune-modulatory interventions must balance the suppression of pathogenic inflammation with the preservation of protective antiviral immune responses. Collectively, these considerations underscore the need for CNS-specific therapeutic approaches that account for the unique cellular environment, immune regulation, and pathological consequences of HIV persistence in glial reservoirs [4].

Collectively, these findings underscore the importance of non-classical cellular reservoirs in HIV-1 neuropathogenesis. Microglia function as latent, reactivation-competent reservoirs within the CNS, whereas astrocytes act as critical immune and metabolic modulators that amplify neuroinflammatory signaling. The coordinated contributions of these glial populations establish a CNS microenvironment characterized by persistent immune activation, stable viral latency, and ongoing neuronal vulnerability. This integrated framework links HIV-1 latency, immune regulation, chronic neuroinflammation, and therapeutic limitations to the persistence of HAND, reinforcing the CNS as a critical landscape of HIV-1 expression and immune modulation [4,60].

## 5. Non-Canonical Cell Types Associated with HIV Persistence

HIV-1 persistence is classically attributed to infection of CD4+ T lymphocytes and myeloid-lineage cells, including monocytes, macrophages, dendritic cells, microglia, and astrocytes, which together constitute the best-characterized cellular reservoirs sustaining viral latency and rebound during ART [25]. Additionally, follicular dendritic cells (FDCs) within lymph node germinal centers represent an important long-lived reservoir of infectious HIV-1. Rather than being productively infected, FDCs capture and retain immune-complexed virions on their surface through Fc and complement receptors, preserving viral infectivity for extended periods. This retained virus can be transferred to susceptible CD4+ T cells, thereby contributing to viral persistence and maintenance of the infectious pool even during suppressive ART [25]. Productive HIV-1 infection classically requires engagement of the CD4 receptor together with the CCR5 or CXCR4 co-receptors, which limits robust viral replication primarily to CD4+ T lymphocytes and certain myeloid lineage cells.

However, despite effective systemic viral suppression, HIV-1 persists in anatomical compartments and cellular populations not traditionally regarded as canonical targets of productive infection, suggesting a broader and more complex reservoir landscape than previously appreciated. These non-canonical cell types are not considered classical replication-competent HIV reservoirs and are discussed here primarily in the context of viral interaction, transient viral carriage, or indirect contributions to viral persistence.

Emerging evidence indicates that several non-canonical cell types can harbor viral material or contribute indirectly to viral persistence through cell-associated viral retention, trans-infection, or reservoir replenishment mechanisms [79,80] (Figure 1). These include megakaryocytes and hematopoietic progenitor cells, which have been implicated as potential sites of proviral integration and persistence under suppressive ART. In contrast, platelets—being anucleated cell fragments—do not support integrated proviral latency but may function as transient carriers of viral material, potentially contributing to viral sequestration, immune modulation, and potential viral transport within the circulation (Figure 1) [79,80]. However, the extent to which platelet-associated HIV represents a true reservoir versus transient viral carriage remains under active investigation. In parallel, endothelial and epithelial cells across multiple tissues, including the vasculature, gastrointestinal tract, renal system, and genital mucosa, have been shown to interact with HIV-1, either by facilitating cell-to-cell viral transfer or serving as long-lived tissue-associated reservoirs under suppressive ART [80,81,82].

Collectively, these non-canonical cellular reservoirs expand the cellular and anatomical framework of HIV persistence and underscore the importance of considering cell-type-specific microenvironments, immune interactions, and trafficking pathways in understanding long-term viral maintenance. Recognition of these alternative reservoirs has significant implications for HIV pathogenesis and cure-oriented strategies, as they may contribute to viral rebound, tissue-specific inflammation, and incomplete eradication despite effective ART. In this section, we highlight selected non-canonical cellular reservoirs and tissue-associated cell types that contribute to HIV-1 persistence through mechanisms including viral sequestration, restricted infection, immune modulation, and cell-to-cell transmission.

### 5.1. Platelets and Megakaryocytes

Platelets are anucleated cellular fragments derived from megakaryocytes that play essential roles in hemostasis and immune regulation. In the context of HIV-1 infection, platelets have been shown, both in vitro and in vivo, to confine HIV within intracellular compartments analogous to the virus-containing compartments described in HIV-infected myeloid cells [82]. These structures are distinct from the classical endosomal-lysosomal system and may protect virions from extracellular immune detection and degradation. Because platelets lack nuclei, they appear to provide a transient intravascular shelter for HIV, capable of carrying viral RNA and potentially transporting intact virions within the circulation, rather than supporting reverse transcription, proviral integration, or productive viral replication within platelets [82].

Using quantitative polymerase chain reaction (PCR) and fluorescence in situ hybridization (FISH)-flow cytometry approaches, HIV-associated replication components have been detected in platelets isolated from ART-treated individuals [82]. Notably, patients with undetectable plasma viral loads exhibited an average of 9.92 HIV RNA copies per million platelets with a 95% confidence interval (CI) of 6.5–17.8, whereas patients with detectable viremia showed substantially higher levels, averaging 122.3 HIV RNA copies per million platelets (95% CI, 65–1111). Importantly, the majority of these individuals had maintained plasma viral suppression below the limit of detection for a median of 26 months prior to sampling, supporting the concept that platelet-associated HIV may persist transiently in the absence of active replication. In addition to viral sequestration, in vitro studies indicate that platelets containing HIV virions can transfer infectious virus to susceptible target cells, supporting cell-to-cell viral transmission, and their association with HIV may originate during thrombopoiesis in infected megakaryocytes within the bone marrow, suggesting that platelet-associated viral carriage may originate from upstream infection within the megakaryocytic lineage and contribute to HIV-associated thrombocytopenia [79,82,83].

Megakaryocytes, the bone marrow-derived precursors of platelets, represent a plausible cellular source of platelet-associated HIV. Megakaryocytes originate from hematopoietic stem and progenitor cells and undergo terminal differentiation to generate platelets. Although platelets themselves are anucleate and incapable of supporting productive viral replication, the presence of HIV RNA and proteins within platelets suggests that they are derived from infected megakaryocytes or earlier progenitor stages during megakaryopoiesis [84].

A key antiviral restriction factor expressed in megakaryocytes and their progenitors is interferon-induced transmembrane protein 3 (IFITM3), which limits viral infectivity by impairing membrane fusion through virion binding and reducing membrane fluidity [85].

HIV infection has been shown to decrease IFITM3 expression in mature megakaryocytes in vitro, with infected cells displaying significantly reduced IFITM3 levels compared to their non-infected counterparts. Notably, this downregulation also affects bystander cells within infected cultures, suggesting a broader virus-induced modulation of the megakaryocytic compartment [84]. Experimental studies using CD34+ cord blood-derived hematopoietic stem and progenitor cells and the megakaryoblastic cell line MEG-01 demonstrated that silencing IFITM3 significantly increased susceptibility to HIV-1 infection [84].

Collectively, these findings suggest that megakaryocytes may represent a potential non-canonical compartment and could contribute indirectly to viral persistence by generating platelets that harbor viral material. This platelet-megakaryocyte axis may represent an underappreciated pathway contributing to HIV dissemination and immune evasion during ART, with potential implications for reservoir maintenance and HIV-associated pathology.

### 5.2. Endothelial Cells

Endothelial cells line the interior surfaces of blood and lymphatic vessels and play critical roles in vascular homeostasis, immune cell trafficking, and tissue-specific immune regulation. Although endothelial cells are not typically considered classical HIV-1 reservoirs, accumulating evidence indicates that they contribute to viral transmission and long-term persistence by facilitating cell-to-cell viral transfer and enhancing the susceptibility of adjacent immune cells [80,86].

In vitro, resting CD4+ T cells are generally resistant to HIV-1 infection; however, in vivo, these cells become susceptible within specific tissue microenvironments. One mechanism underlying this discrepancy involves interactions between integrins expressed on CD4+ T cells, such as lymphocyte function-associated antigen 1 (LFA-1) and very late antigen 4 (VLA-4), as well as their corresponding endothelial ligands, intercellular adhesion molecule 1 (ICAM-1) and vascular cell adhesion molecule 1 (VCAM-1) [87,88]. Through these integrin-dependent interactions, endothelial cells can capture HIV-1 virions and promote transinfection, transferring the virus to resting CD4+ T cells without themselves supporting productive infection [80]. To date, there is limited evidence supporting productive or latent HIV-1 infection in endothelial cells under physiological conditions. In addition, engagement of adhesion pathways further enhances the susceptibility of these target cells to productive HIV infection, thereby increasing infection efficiency and potentially facilitating the seeding or maintenance of viral reservoirs [80,88]. 

Beyond facilitating transinfection, endothelial activation has broader implications for HIV-associated pathology. Elevated VCAM-1 expression has been linked to increased cardiovascular risk, including stroke and atherosclerosis, in individuals living with HIV [87]. ART can reduce circulating VCAM-1 levels, but endothelial dysfunction may persist due to chronic inflammation and ART-associated vascular effects, underscoring the complex interplay between viral persistence, immune activation, and vascular health.

Intestinal endothelial cells, also referred to as human intestinal microvascular endothelial cells (HIMECs), are of particular relevance due to their localization within gut-associated lymphoid tissue (GALT), which harbors the largest reservoir of CD4+ T cells in the body [88]. Within the GALT, HIMECs are in constant contact with CD4+ T cells and secrete cytokines such as IL-6, which enhances cellular susceptibility to HIV infection without inducing full T-cell activation [88,89]. Overall, these interactions create conditions that may facilitate productive infection and latency establishment in nearby CD4+ T cells, contributing to viral persistence in the intestinal mucosa, although the precise mechanisms and their in vivo significance remain under ongoing investigation.

A major CD4+ T-cell subset within the GALT is the Th17 population, which plays a critical role in maintaining intestinal barrier integrity by secreting interleukin-17A (IL-17A) [88,90]. IL-17A promotes tight junction protein expression and antimicrobial peptide production, reinforcing mucosal defenses. However, IL-17A also acts synergistically with TNF on intestinal endothelial cells to induce chemokines such as C-C motif chemokine ligand 20 (CCL20), which recruits CCR6+ Th17 cells to sites of inflammation [88,90]. During HIV exposure, increased CCL20 production by activated intestinal endothelial cells leads to preferential accumulation of Th17 cells, creating a microenvironment that enhances HIV transinfection and infection efficiency.

Collectively, endothelial-T-cell interactions within vascular and mucosal tissues establish permissive microenvironments that facilitate HIV transmission, latency establishment, and reservoir maintenance. Although endothelial cells do not support robust productive infection, their role as facilitators of viral spread and immune cell susceptibility positions them as key contributors to HIV persistence during ART.

### 5.3. Epithelial Cells

Epithelial cells line organ surfaces and mucosal barriers throughout the body and play critical roles in tissue integrity, immune defense, and pathogen exclusion. Although epithelial cells are not considered classical targets of productive HIV-1 infection, growing evidence indicates that specific epithelial populations can contribute to viral persistence, compartmentalized replication, and cell-to-cell transmission under certain pathological conditions [91].

In the kidney, renal tubular epithelial (RTE) cells have emerged as a potential non-canonical cell associated with HIV-1 persistence. Clinical evidence from kidney transplant recipients has demonstrated the presence of HIV-1 strains in urine that are genetically distinct from circulating blood-derived virus, indicating localized viral production within renal tissue [91,92]. During HIV-associated nephropathy, inflammatory conditions promote close interactions between infected macrophages and RTE cells, facilitating viral transfer through cell-to-cell contact [91]. Once infected, RTE cells can support clonal amplification of integrated proviral DNA, a mechanism well characterized in CD4+ T cells, whereby proviruses integrated near host genes are replicated during cell division, contributing to long-term viral persistence [91,93]. Notably, infected RTE cells have been shown to produce infectious virus capable of infecting lymphoid cells, including T cells and monocytes, thereby linking renal epithelial infection to systemic viral dissemination [91].

Epithelial cells of the female reproductive tract also represent a critical interface for HIV transmission and persistence. The endocervix is lined by columnar epithelial cells that secrete mucus and help limit viral penetration, whereas the squamous epithelium of the vagina and ectocervix is more permissive to viral exposure [94]. Microabrasions generated during sexual intercourse can disrupt epithelial integrity, enabling paracellular penetration of HIV between epithelial cells following tight junction alteration [81]. Although this route of transmission is relatively inefficient, squamous epithelial cells can capture and retain HIV virions, acting as transient carriers that facilitate subsequent transfer to susceptible CD4+ target cells [94].

Importantly, the presence of HIV DNA and RNA in genital secretions has been detected even in individuals receiving suppressive ART, underscoring the potential for epithelial-associated viral persistence and transmission despite systemic viral control [95]. Collectively, these findings indicate that while epithelial cells do not support robust productive infection, their capacity to harbor viral material, undergo clonal expansion, and mediate cell-to-cell transmission positions them as functionally significant contributors to HIV persistence across tissue compartments.

## 6. HIV-1 Latency and Reactivation Mechanisms

A defining feature of HIV-1 persistence is the establishment of proviral latency following integration into the host genome. Although integration is an obligatory step in the viral life cycle, accumulating evidence demonstrates that the genomic and epigenetic context in which integration occurs profoundly influences subsequent viral transcriptional activity, inducibility, and long-term reservoir stability. In this section, we discuss how HIV-1 integration site selection, host chromatin features, and nuclear organization shape the transcriptional fate of the provirus and contribute to the functional heterogeneity of latent HIV reservoirs.

### 6.1. Integration Sites

Accumulating evidence demonstrates that HIV-1 integration into the host genome is a highly directed process that critically shapes the transcriptional fate of the provirus and contributes to the heterogeneity of viral latency [96,97,98]. HIV-1 preferentially integrates within introns of actively transcribed genes that are enriched in open chromatin and epigenetic marks associated with transcriptional elongation, most notably histone H3 lysine 36 trimethylation (H3K36me3) [96,97,99,100]. These integration events are spatially organized within accessible nuclear compartments, frequently positioned near the nuclear pore complex, which facilitates nuclear entry and targeting of the viral pre-integration complex [97,100,101].

Integration into transcriptionally active regions has been consistently observed across experimental models and primary cells from individuals living with HIV, including proviruses detected early after the initiation of ART [102]. Recurrent integration into specific host genes, such as *BACH2*, *MKL2*, and *STAT5B*, has been documented, suggesting that certain genomic loci not only favor viral insertion but also support clonal expansion and long-term persistence of infected cells [97,100,101]. Targeting of these genomic regions is mediated by host cofactors, including cleavage and polyadenylation specificity factor 6 (CPSF6), which interacts with the viral capsid to guide the pre-integration complex toward euchromatic regions, and lens epithelium-derived growth factor (LEDGF), which tethers viral integrase to H3K36me3-marked nucleosomes, thereby promoting integration within actively transcribed gene bodies [96,97].

Importantly, integration into active genes does not uniformly result in productive viral expression, and its functional outcome is highly dependent on cellular context and transcriptional state, particularly in resting CD4+ T cells [97,99,103]. In these cells, host gene transcription can impose transcriptional interference at the viral 5′ LTR, suppressing viral initiation and generating nonfunctional host-viral hybrid transcripts that stabilize latency [96,104]. Conversely, a substantial fraction of proviruses integrates into non-genic, pseudogenic, or repetitive genomic regions, including satellite DNA, which are associated with minimal transcriptional activity and deep latency [100]. Single-provirus analyses have demonstrated that viral expression correlates strongly with the transcriptional and epigenetic state of the host locus, underscoring integration site context as a key determinant of whether proviruses remain inducible or transcriptionally silent [100,103]. Overall, these findings position integration site selection as a central regulator of HIV-1 expression and a major contributor to the functional diversity of the latent reservoir.

### 6.2. Transcriptional Blocks

At the transcriptional level, HIV-1 expression is governed by a series of post-initiation regulatory checkpoints that play a central role in the establishment and maintenance of viral latency [105,106,107]. Unlike irreversible silencing at transcriptional initiation or through transcriptional interference, these regulatory blocks are largely reversible and occur during early transcriptional elongation, polyadenylation, and RNA splicing.

**Figure 4:**
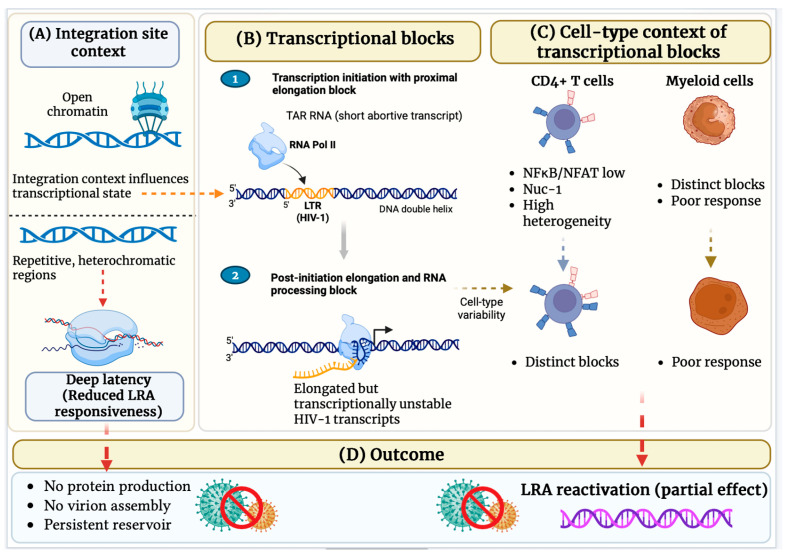
Integration site context and transcriptional blocks shape HIV-1 latency and cell-type-specific responsiveness to latency-reversing agents. (**A**) Integration site context. HIV-1 integration into transcriptionally active open chromatin favors a more permissive transcriptional environment, whereas integration within repetitive or heterochromatic regions promotes deeper proviral silencing. This repressive chromatin context is associated with deep latency, characterized by reduced responsiveness to latency-reversing agents (LRAs) and minimal or absent productive viral replication, thereby supporting long-term reservoir persistence. (**B**) Transcriptional blocks. Following integration, HIV-1 transcription is limited by multiple post-integration regulatory checkpoints. (1) During transcription initiation, RNA polymerase II (RNA Pol II) frequently undergoes proximal pausing, producing short TAR-containing abortive transcripts. (2) Additional restrictions occur at post-initiation elongation and RNA processing steps, generating elongated but transcriptionally unstable HIV-1 transcripts. Together, these mechanisms maintain low viral gene expression despite proviral integration. (**C**) Cell-type context of transcriptional blocks. The strength and nature of transcriptional restrictions vary by cellular reservoir. In resting cluster of differentiation **4** (CD4+) T cells, limited nuclear factor kappa B and nuclear factor of activated T-cell (NF-κB/NFAT) activity, nucleosome-1 (Nuc-1) positioning, and cellular heterogeneity contribute to reversible latency. In myeloid cells, including microglia/macrophage-lineage cells, distinct transcriptional constraints and reduced responsiveness to LRAs further stabilize viral persistence. (**D**) Outcome. The combined effects of integration site selection, transcriptional repression, and cell-type-specific regulation result in minimal viral protein production, lack of virion assembly, and maintenance of a persistent latent reservoir. Pharmacologic LRA stimulation produces only partial reactivation, particularly in deeply latent or myeloid reservoirs. Solid arrows indicate the direction of molecular progression, whereas dashed arrows denote conceptual relationships or cell-type-dependent variability in the establishment of transcriptional blocks and outcomes. Created with BioRender.com.

One of the most prominent barriers to productive HIV-1 transcription arises during early elongation, where RNA polymerase II (RNAP II) pauses shortly after initiation (Figure 4). In resting CD4+ T cells, this elongation block prevents efficient synthesis of full-length viral transcripts and leads to the accumulation of short, abortive RNAs [106,108]. Efficient transcriptional elongation requires the viral transactivator protein Tat, which binds to the transactivation response (TAR) element located at the 5′ end of all nascent HIV transcripts. By recruiting host elongation factors, Tat enhances RNAP II processivity and enables productive transcription. Consistent with this mechanism, an increased abundance of TAR-containing transcripts relative to full-length LTR transcripts serves as a molecular indicator of impaired transcriptional elongation in latently infected cells [106].

Beyond elongation, additional reversible blocks occur at the levels of polyadenylation and RNA splicing, both of which are essential for transcript stability, nuclear export, and efficient translation. Defective polyadenylation limits transcript maturation, while inefficient splicing restricts the production of multiply spliced viral RNAs required for expression of key regulatory and structural proteins [109]. The viral Rev protein plays a critical role in overcoming post-transcriptional restrictions by mediating the nuclear export of unspliced and partially spliced HIV RNAs, thereby enabling their translation in the cytoplasm [110].

Collectively, these post-initiation transcriptional and post-transcriptional blocks cooperate to stabilize HIV-1 latency, allowing integrated proviruses to persist in transcriptionally silent or low-expression states even within chromatin environments that are otherwise permissive for gene expression.

### 6.3. Latency-Reversing Agents (LRAs)

HIV-1 latency is maintained primarily within T-cell central memory (TCM) and effector memory (TEM), as well as select myeloid populations, where the viral promoter is embedded within a restrictive chromatin environment. This state is characterized by the presence of repressive nucleosomes, including nucleosome-1 (Nuc-1), and histone modifications such as deacetylation and methylation that limit transcription factor recruitment and RNAP II processivity (Figure 4) [96,99,102,104]. In addition to epigenetic repression, post-initiation transcriptional blocks, encompassing early elongation, polyadenylation, and RNA splicing, further restrict the production of full-length viral transcripts, reinforcing latency even within chromatin regions that are otherwise transcriptionally active [111,112].

The functional heterogeneity of the latent reservoir is strongly influenced by the genomic context of proviral integration. Proviruses integrated within introns of actively transcribed genes and euchromatic regions exhibit greater inducibility, whereas those located in non-genic, pseudogenic, or repetitive genomic regions display minimal transcriptional activity and deep latency [96,99,100,103]. These differences underscore the challenge of uniformly reactivating the latent reservoir across diverse cellular and epigenetic contexts.

LRAs have been developed to overcome epigenetic and post-initiation barriers to HIV-1 transcription. Histone deacetylase inhibitors (HDACis), including vorinostat, romidepsin, and panobinostat, promote histone acetylation and remodeling of the Nuc-1 nucleosome, thereby increasing the initiation and elongation of viral transcripts [96,97,103]. Histone methyltransferase inhibitors (HMTis) counteract repressive H3K9 and H3K27 methylation marks, while BAF complex inhibitors reduce the occupancy of repressor nucleosomes at the viral promoter, facilitating transcriptional elongation [97,99,111,112].

Additional LRA classes target transcriptional elongation through distinct mechanisms. Bromodomain and extraterminal domain inhibitors (BETis), such as JQ1 and iBET, displace BRD4 and liberate positive transcription elongation factor b (P-TEFb), thereby enhancing elongation at proviruses integrated within active gene regions [104]. Protein kinase C (PKC) agonists, including prostratin, ingenol derivatives, and bryostatin-1, activate NF-κB signaling and promote the generation of fully processed viral transcripts, with more pronounced effects in effector memory T cells compared to TCMs. In parallel, TLR agonists and benzotriazoles enhance STAT5 recruitment to the viral promoter, thereby relieving elongation and splicing blocks in primary cells [97,104,113,114].

Despite these advances, LRA-induced reactivation remains incomplete and heterogeneous, affecting only a fraction of the latent reservoir (Figure 4). Reactivation efficiency varies by cell type, differentiation state, integration site, epigenetic context, and prior antiretroviral exposure, underscoring the limitations of single-agent approaches. These observations emphasize the need for rational LRA combinations that target multiple regulatory layers to achieve more consistent and effective reactivation of latent HIV reservoirs.

## 7. Host Restriction Factors and Intrinsic Cellular Defenses in HIV-1 Infection

Host cells possess intrinsic defense mechanisms that act within infected cells to restrict viral replication and limit pathogen spread. In the context of HIV-1 infection, these defenses operate independently of adaptive immunity and include a diverse group of host restriction factors that interfere with discrete stages of the viral life cycle. These cell-intrinsic antiviral mechanisms are particularly important in HIV-1-susceptible populations such as CD4+ T lymphocytes, monocytes, macrophages, and dendritic cells, where they contribute to viral suppression, latency establishment, and reservoir persistence [115,116]. Several of these restriction mechanisms intersect with innate sensing and inflammatory pathways discussed in earlier sections of this review, highlighting how shared antiviral strategies operate across multiple HIV-susceptible cell types.

**Figure 5:**
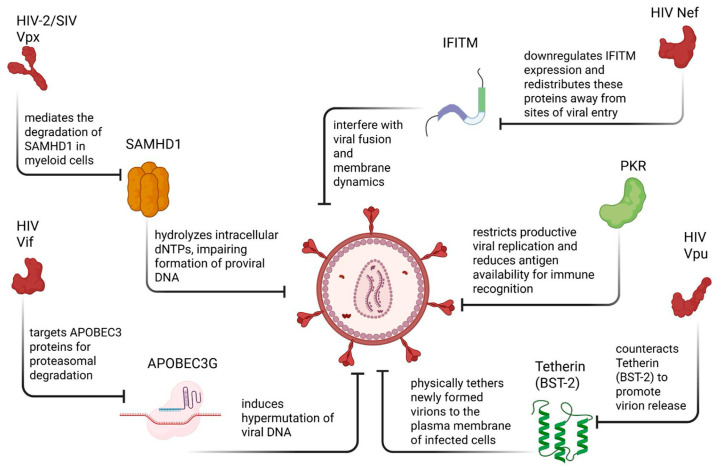
Host restriction factors and viral countermeasures regulating HIV replication. Host cells possess intrinsic antiviral defense mechanisms that restrict HIV replication at multiple stages of the viral life cycle. Several host restriction factors interfere with viral entry, reverse transcription, genome integrity, protein synthesis, and virion release. Sterile alpha motif and histidine-aspartate domain-containing protein 1 (SAMHD1) limits proviral DNA formation in myeloid cells by hydrolyzing intracellular deoxynucleoside triphosphates (dNTPs), thereby restricting efficient reverse transcription. Apolipoprotein B mRNA editing enzyme catalytic subunit 3G (APOBEC3G) induces cytidine deamination during reverse transcription, resulting in G-to-A hypermutations that destabilize the viral genome and generate defective proviruses. Interferon-induced transmembrane proteins (IFITMs) inhibit viral entry by interfering with membrane fusion and viral trafficking. Protein kinase R (PKR) restricts viral replication by promoting translational arrest through phosphorylation of eIF2α and reducing viral protein synthesis. Tetherin (BST-2) prevents the release of newly formed virions by physically tethering budding particles to the plasma membrane of infected cells. HIV has evolved accessory proteins that counteract these host defenses. Vif promotes proteasomal degradation of APOBEC3 proteins, while Vpu antagonizes Tetherin to restore virion release. Nef downregulates IFITM expression and redistributes these proteins away from viral entry sites. Vpx, encoded by HIV-2 and several simian immunodeficiency viruses (SIV), mediates the degradation of SAMHD1 in myeloid cells, relieving nucleotide restriction and facilitating viral replication. Together, these interactions illustrate the dynamic molecular arms race between host intrinsic restriction factors and viral antagonistic mechanisms that shape HIV replication, persistence, and immune evasion. Image created with Biorender.com.

Host restriction factors function by targeting essential viral processes, including reverse transcription, integration, transcription, virion assembly, and viral release. Many of these proteins are constitutively expressed or induced by IFNs, positioning them as key effectors of innate antiviral immunity. Prominent restriction factors involved in HIV-1 control include SAMHD1, APOBEC3 family members, Tetherin (BST-2), PKR, and interferon-induced transmembrane (IFITM) proteins (Figure 5). Through distinct molecular mechanisms, these factors reduce viral replication efficiency, promote the formation of defective proviral genomes, or limit the production and spread of infectious virions [117,118,119,120,121].

The effectiveness of host restriction factors has driven the evolution of viral countermeasures (Figure 5). HIV-1 encodes several accessory proteins, including Vif, Vpu, Nef, and Vpr, that antagonize or bypass host restriction mechanisms. For example, Vif targets APOBEC3 proteins for proteasomal degradation, while Vpu counteracts Tetherin (BST-2) to promote virion release. In contrast, Vpx, encoded by HIV-2 and many simian immunodeficiency viruses (SIV) but not by HIV-1, mediates the degradation of SAMHD1 in myeloid cells, thereby relieving dNTP restriction. The dynamic interplay between host restriction factors and viral antagonists represents a central molecular arms race that shapes viral fitness, immune activation, and long-term persistence within infected individuals [121,122,123].

Importantly, restriction factor activity does not uniformly eliminate HIV-1 but instead contributes to heterogeneous infection outcomes, including partial suppression of viral replication, establishment of transcriptionally silent proviruses, and maintenance of long-lived viral reservoirs. As such, host restriction factors occupy a critical position at the intersection of innate immunity, viral latency, and therapeutic intervention. Although these mechanisms are described individually below, many of them operate concurrently across different cellular reservoirs, reflecting common antiviral strategies that shape HIV persistence in diverse cellular environments.

In this section, we focus on key host restriction factors and their associated signaling pathways that regulate HIV-1 replication, latency, and immune evasion (Figure 5), highlighting both their antiviral mechanisms and the viral strategies that counteract them.

### 7.1. SAMHD1 and Control of Proviral DNA Formation

SAMHD1 is a well-characterized host restriction factor that limits HIV-1 replication in dendritic cells, macrophages, and resting CD4+ T cells. SAMHD1 restricts infection primarily by hydrolyzing intracellular dNTPs, thereby reducing the nucleotide pool required for efficient reverse transcription of the viral RNA genome. Similar restriction processes operating in myeloid populations have been discussed in the context of macrophage and microglial antiviral responses earlier in this review [117,121,122].

In dendritic cells and macrophages, where SAMHD1 expression is high, this restriction contributes to inefficient reverse transcription and reduced viral replication. Beyond its direct antiviral activity, SAMHD1 also influences innate immune sensing. When SAMHD1-mediated restriction is relieved, either through viral antagonism or experimental depletion, accumulation of viral DNA intermediates can activate DNA-sensing pathways, including the stimulator of interferon genes (STING) axis, leading to type I IFN production and NF-κB activation [116,117,123]. These signaling events further shape inflammatory responses and antigen presentation, linking SAMHD1 activity to both antiviral defense and immune modulation.

### 7.2. APOBEC3G Restriction of HIV-1 Genome Integrity

APOBEC3G is an antiviral cytidine deaminase that restricts HIV-1 replication by inducing hypermutation of viral DNA during reverse transcription. In non-permissive cells expressing APOBEC3G, the enzyme is incorporated into assembling virions and subsequently deaminates cytosine residues in the minus-strand viral DNA, leading to G-to-A hypermutations. These mutations destabilize the viral genome, resulting in defective proviruses that are unable to produce functional viral proteins [117,121,122,123].

The viral protein Vif is essential for neutralizing APOBEC3G-mediated restriction. Vif promotes APOBEC3G degradation, thereby restoring viral infectivity. Host factors modulate this interaction: proteins such as MDM2 promote Vif degradation and enhance APOBEC3G antiviral activity, whereas CBF-β stabilizes Vif and suppresses APOBEC3G function. Additionally, phosphorylation of Vif by AKT enhances its stability and ability to counteract APOBEC3G, while inhibition of AKT destabilizes Vif and increases APOBEC3G-mediated restriction [117,122,124].

Importantly, APOBEC3G activity contributes not only to restriction of active replication but also to the accumulation of genetically defective proviruses within viral reservoirs. These defective genomes may still express viral RNA or proteins, contributing to chronic immune activation despite suppressive ART.

### 7.3. Tetherin-Mediated Inhibition of Virion Release

Tetherin (BST-2) is an IFN-induced restriction factor that inhibits HIV-1 dissemination by physically tethering newly formed virions to the plasma membrane of infected cells. This mechanism prevents efficient viral release and reduces cell-free viral spread. Structural features of Tetherin are critical for its antiviral function, and overexpression of Tetherin strongly inhibits virion secretion in the absence of viral antagonists [117,124,125,126].

HIV-1 counteracts Tetherin primarily through the accessory protein Vpu, which downregulates Tetherin from the cell surface and sequesters it within intracellular compartments such as the trans-Golgi network. By preventing Tetherin localization at viral assembly sites, HIV-1 restores efficient virion release and enhances viral transmission. In addition to its role in restricting viral egress, Tetherin can activate innate immune signaling pathways, including NF-κB, linking virion retention to inflammatory responses that may influence reservoir persistence [117,124,125,126,127].

### 7.4. PKR as a Critical Host Factor in Antiviral Defense and HIV-1 Persistence

PKR is a central component of the host’s intrinsic antiviral defense and functions as both an IFN-stimulated gene and a pattern recognition receptor. As discussed in earlier sections in the context of innate immune responses in myeloid and T-cell populations, PKR activation represents a shared antiviral mechanism across multiple HIV-susceptible cell types. PKR is activated upon recognition of dsRNA, a molecular signature commonly generated during viral replication [128,129,130]. Upon activation, PKR acts as a key sensor linking viral detection to translational control, innate immune signaling, and cellular stress responses.

Activation of PKR occurs through dimerization and autophosphorylation, a process facilitated by the PKR-activating protein (PACT). Once activated, PKR phosphorylates eIF2α, leading to global translational arrest. This inhibition of protein synthesis limits viral replication by preventing the production of viral proteins while simultaneously promoting the formation of stress granules, which function as regulatory hubs for antiviral signaling [128,129,130,131]. These stress granules contribute to the coordination of innate immune responses and can modulate PKR activity itself, reinforcing translational repression during infection.

In the context of HIV-1 infection, PKR can restrict productive viral replication and reduce antigen availability for immune recognition. At the same time, sustained PKR signaling may promote cellular survival through autophagy and limit excessive immune activation, thereby creating conditions that favor viral persistence rather than clearance [17,131,132,133]. This dual role positions PKR as a molecular checkpoint that can both restrict viral protein synthesis and contribute to the maintenance of low-level or latent infection states under certain cellular conditions.

Beyond translational control, PKR also interfaces with broader innate immune signaling pathways. Activated PKR can induce IFN responses and stimulate inflammatory signaling cascades, including NF-κB and IκB kinase (IKK) activation, thereby amplifying antiviral gene expression and shaping the inflammatory microenvironment [128,132,133,134]. Through these mechanisms, PKR integrates viral sensing, translational repression, and immune signaling, influencing both antiviral defense and the balance between viral clearance and persistence during HIV-1 infection.

### 7.5. IFITM Restriction of HIV-1 Entry and Viral Counteraction by Nef

The HIV-1 accessory protein Nef is among the earliest and most abundantly expressed viral proteins during infection and plays a critical role in viral pathogenesis. Nef is a multifunctional protein of approximately 200–215 amino acids that enhances viral replication and immune evasion by downregulating host surface molecules, including CD4 and MHC-I, thereby reducing immune recognition of infected cells. In addition, Nef modulates signaling pathways in T cells and macrophages and perturbs actin filament dynamics within the cytoskeleton, facilitating viral replication and cell-to-cell transmission. Notably, Nef can be detected in patient serum even when plasma HIV-1 RNA levels are suppressed, underscoring its persistence and functional relevance during infection [123,124,135,136].

IFITMs are host restriction factors that inhibit viral infection primarily at the entry stage. IFITM proteins localize to cellular membranes, including the plasma membrane and endosomal compartments, where they interfere with viral fusion and membrane dynamics. In the context of HIV-1, IFITMs restrict viral entry in a co-receptor-dependent manner, with differential effects observed for CCR5- and CXCR4-tropic viruses. By altering membrane fluidity and fusion competence, IFITMs reduce the efficiency of viral entry into target cells and limit subsequent replication [124,137,138,139].

HIV-1 counteracts IFITM-mediated restriction through Nef-dependent mechanisms. Nef downregulates IFITM expression and redistributes these proteins away from sites of viral entry, thereby alleviating fusion inhibition and restoring infectivity. Through this antagonism, Nef enables HIV-1 to overcome IFN-induced antiviral barriers while maintaining efficient entry and spread. Collectively, the interplay between IFITM restriction and Nef-mediated counteraction highlights a critical host–virus interface at the level of viral entry and underscores how HIV-1 subverts intrinsic immune defenses to promote persistence [85,124,139].

## 8. Inflammatory Microenvironments and Intercellular Crosstalk in HIV Persistence

HIV-1 persistence is not determined solely by intrinsic properties of infected cells but is profoundly shaped by the surrounding inflammatory microenvironment and dynamic interactions between infected and uninfected immune populations. Chronic immune activation, sustained cytokine production, and cell–cell communication collectively create permissive niches that stabilize viral latency, facilitate episodic reactivation, and contribute to long-term reservoir maintenance [140,141].

Following infection, HIV-1 preferentially establishes latency within memory CD4+ T cells, which retain migratory capacity and circulate between peripheral tissues and secondary lymphoid organs. This mobility enables infected cells to access diverse tissue microenvironments while remaining shielded from immune clearance. Within lymphoid tissues, prolonged cell–cell contacts promote efficient viral dissemination through virological synapses, enhancing infection efficiency beyond that achieved by cell-free virus [140,142]. Importantly, transitions between activated and quiescent states allow infected T cells to alternate between productive replication and transcriptional silencing, reinforcing reservoir stability.

Chronic HIV infection is characterized by persistent inflammation driven by viral replication, microbial translocation, and innate immune sensing. Engagement of pattern recognition receptors, including TLRs, such as TLR2, TLR7, and TLR9, activates downstream signaling pathways, including NF-κB and type I IFN responses, thereby sustaining the production of inflammatory mediators, including IL-1β, TNF-α, IFN-α/β, and chemokines such as IP-10 [143,144]. While these pathways initially contribute to antiviral defense, prolonged activation paradoxically promotes immune exhaustion, tissue damage, and conditions favorable for viral persistence.

Innate immune sensing also contributes to reservoir dynamics by activating inflammasomes. Cytosolic DNA sensors such as interferon gamma-inducible protein 16 (IFI16) initiate inflammasome assembly via apoptosis-associated speck-like protein containing a CARD (ASC) and caspase-1, leading to pyroptotic cell death in bystander CD4+ T cells. This process contributes to CD4+ T-cell depletion while simultaneously driving inflammation that sustains residual reservoirs and promotes viral spread [143,145]. Thus, cell death and immune activation become tightly linked processes that paradoxically support HIV persistence.

Within secondary lymphoid tissues, structural and cellular organization further amplifies these effects. Lymph nodes provide specialized niches enriched in cytokines, stromal networks, and APCs that promote prolonged interactions among immune cells. Subcapsular sinus macrophages, fibroblastic reticular cells, and dendritic cells coordinate antigen presentation and cytokine gradients, creating environments that favor cell–cell transmission and reservoir seeding [140,146]. Homeostatic cytokines such as IL-7 and IL-15 support survival and low-level proliferation of latently infected memory T cells, enabling long-term maintenance of the reservoir even under suppressive ART [141].

Intercellular crosstalk extends beyond lymphoid tissues to peripheral and CNS compartments. In the CNS, inflammatory signaling between infected myeloid cells, glial cells, and infiltrating lymphocytes contributes to neuroinflammation and HAND. Cytokines and viral proteins released by infected cells can disrupt neuronal homeostasis and perpetuate immune activation independently of productive viral replication, illustrating how microenvironmental signals uncouple viral expression from systemic viremia [144].

Collectively, these findings highlight that HIV persistence is sustained not only by intrinsic viral and cellular mechanisms but also by inflammatory microenvironments and intercellular signaling networks that reinforce latency, immune evasion, and tissue-specific pathogenesis. Understanding how these contextual factors regulate HIV expression across diverse cellular landscapes is essential for developing therapeutic strategies to disrupt reservoir stability and achieve durable viral remission [140,144].

## 9. Natural Immunomodulators and Therapeutics Prospects

Natural compounds derived from plants and medicinal mushrooms have attracted increasing attention for their capacity to modulate HIV-1 replication, transcriptional activity, and immune regulation via host-directed mechanisms. Unlike conventional ART, which primarily targets viral enzymes such as reverse transcriptase, integrase, and protease, many bioactive natural compounds modulate cellular signaling pathways that govern HIV gene expression, inflammatory responses, and immune cell activation states.

This host-directed mode of action is particularly relevant in the context of HIV persistence, as viral latency and restricted replication are tightly regulated by host transcription factors, chromatin architecture, and immune signaling networks. Cellular pathways involving NF-κB, STAT signaling, histone deacetylases (HDACs), sirtuins, oxidative stress responses, and pattern recognition receptors play central roles in determining whether HIV remains transcriptionally silent or becomes reactivated. Natural compounds capable of influencing these pathways, therefore, represent valuable tools for probing the molecular regulation of HIV expression and, potentially, for complementing existing therapeutic strategies.

Importantly, natural immunomodulators do not function as direct antiviral agents in the classical sense. Instead, their effects are often pleiotropic, context-dependent, and cell-type specific, influencing both antiviral immunity and inflammatory tone. This duality is particularly relevant in tissues such as the CNS and lymphoid compartments, where excessive immune activation can exacerbate pathology, while insufficient activation can permit viral persistence. As a result, natural compounds have been explored as both potential latency-modulating agents and immunoregulatory adjuncts to reduce chronic inflammation without promoting viral reactivation.

Despite growing interest, the therapeutic application of natural compounds in HIV remains challenging. Many studies are limited to in vitro or ex vivo systems, and issues related to bioavailability, dosing, pharmacokinetics, and off-target effects constrain clinical translation. Nevertheless, mechanistic insights from these compounds have significantly contributed to our understanding of host pathways that regulate HIV transcription, immune evasion, and persistence. In this section, we will focus on some of the selected natural compounds, including polyphenols, flavonoids, and mushroom-derived polysaccharides, providing a valuable framework for examining host-directed regulation of HIV expression and immune modulation.

### 9.1. Quercetin

**Figure 6:**
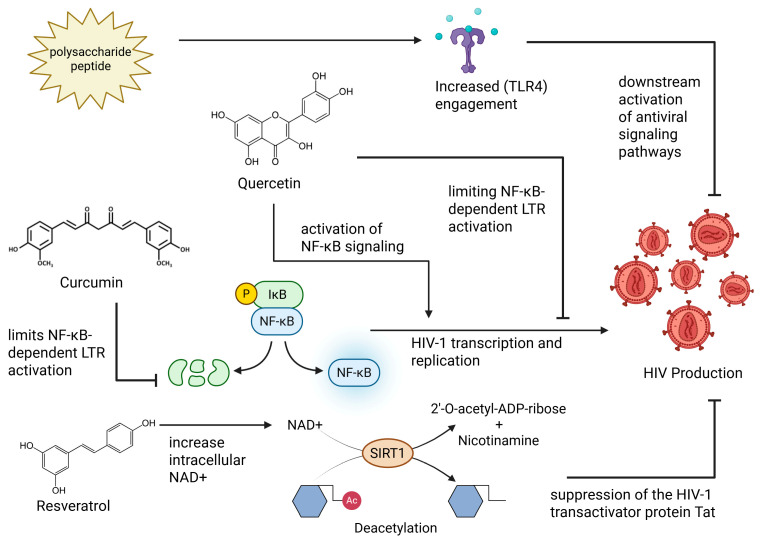
Host-directed immunomodulatory effects of natural compounds on HIV-1 transcription and production. Natural immunomodulators influence HIV-1 expression primarily through regulation of host signaling and epigenetic pathways rather than direct inhibition of viral enzymes. Polysaccharide peptide (PSP) derived from *Coriolus versicolor* enhances Toll-like receptor 4 (TLR4) engagement, promoting downstream activation of antiviral signaling pathways that contribute to a cellular environment less permissive for HIV-1 production. Polyphenolic compounds such as quercetin and curcumin modulate nuclear factor kappa B (NF-κB) signaling, thereby limiting NF-κB-dependent activation of the HIV-1 long terminal repeat (LTR) and reducing viral transcription under specific cellular contexts. Resveratrol increases intracellular nicotinamide adenine dinucleotide (NAD^+^) levels and activates the NAD^+^-dependent deacetylase sirtuin 1 (SIRT1), leading to deacetylation and functional suppression of the HIV-1 transactivator of transcription (Tat). Collectively, these host-directed mechanisms converge on reduced HIV-1 transcriptional activity and viral production. Arrows indicate activation or enhancement of signaling pathways, whereas blunt-ended lines indicate inhibitory effects. Image created with BioRender.com.

Quercetin is a naturally occurring flavonoid abundant in fruits and vegetables and has been widely investigated for its antioxidant, anti-inflammatory, and antiviral properties. In the context of HIV-1 infection, quercetin has been shown to inhibit viral replication in primary human peripheral blood mononuclear cells (PBMCs), as evidenced by reduced levels of the viral capsid protein p24. These findings suggest that quercetin interferes with early or intermediate stages of the viral life cycle and/or modulates host pathways required for efficient viral production. At the molecular level, quercetin suppresses HIV-1 LTR-driven transcription, indicating a direct effect on viral gene expression [147]. This suppressive activity has been linked to modulation of host transcription factors, particularly NF-κB, a central regulator of HIV transcription (Figure 6). By limiting NF-κB-dependent LTR activation, quercetin may reduce viral transcription and virion production during active infection.

Notably, quercetin exhibits context-dependent effects on HIV latency. In cellular models of latent infection, quercetin has been reported to promote HIV-1 reactivation through activation of NF-κB signaling (Figure 6), particularly when used in combination with established LRAs such as valproic acid or prostratin [148]. HIV-1 transcription is tightly regulated at the level of LTR, which contains binding sites for key host transcription factors, including NF-κB, SP1, and AP-1. Among these, NF-κB plays a central role in activating viral gene expression through two conserved κB enhancer elements within the LTR core promoter region [148]. In vitro studies demonstrate that quercetin enhances NF-κB nuclear translocation and transcriptional activity, leading to increased LTR-driven reporter expression. Importantly, quercetin failed to activate LTR constructs lacking NF-κB binding sites, indicating that its effect is NF-κB dependent. Mechanistically, quercetin appears to promote activation of canonical NF-κB signaling pathways, thereby facilitating p65 nuclear localization and enhancer occupancy. These findings suggest that quercetin may function as a latency-modulating agent by promoting transcriptional activation of integrated provirus through NF-κB–mediated mechanisms. However, whether this activation supports controlled latency reversal or enhances productive replication remains context-dependent and requires further investigation.

This dual behavior highlights the complex, dose-dependent nature of quercetin’s activity, underscoring that its effects on HIV expression are influenced by cellular activation state, treatment context, and combinatorial strategies.

While these findings underscore quercetin’s potential as a host-directed modulator of HIV transcription, most evidence to date derives from in vitro and ex vivo systems. As such, further investigation is required to determine the translational relevance, optimal dosing, and therapeutic window of quercetin in the context of HIV infection and latency modulation.

### 9.2. Curcumin

Curcumin is a polyphenolic compound derived from Curcuma longa (turmeric) and is widely recognized for its anti-inflammatory, antioxidant, and antimicrobial properties. In HIV-1 research, curcumin has been investigated primarily for its ability to suppress viral transcription and replication via host-directed regulatory mechanisms rather than solely through direct antiviral activity [149]. One of the best-characterized anti-HIV effects of curcumin involves the proteasomal degradation of the viral Tat. Curcumin promotes ubiquitin-independent degradation of Tat through activation of the 20S proteasomal pathway by competitively inhibiting NADH binding at the NQO1 interaction site. Tat is an intrinsically disordered protein that relies on stabilization by NAD(P)H-bound NQO1 to avoid proteasomal degradation. By disrupting this protective interaction, curcumin facilitates Tat destabilization and degradation, thereby reducing Tat-dependent LTR transcriptional amplification. Tat is essential for efficient transcription from the HIV-1 LTR, and its degradation leads to a marked reduction in viral gene expression and replication. Through this mechanism, curcumin functions predominantly as a transcriptional suppressor, distinguishing it from LRA, which aims to induce proviral expression [150].

In addition to targeting Tat, curcumin modulates host signaling pathways implicated in HIV transcription and immune activation. These include inhibition of NF-κB signaling (Figure 6) and regulation of HDAC-dependent chromatin remodeling, both of which contribute to a transcriptionally restrictive environment for HIV. In addition to targeting Tat stability, curcumin modulates host signaling pathways implicated in HIV-1 transcriptional regulation. Curcumin has been shown to inhibit NF-κB signaling by suppressing IKK activation and reducing p65 nuclear translocation. Because NF-κB binds to two conserved κB enhancer elements within the HIV-1 LTR, inhibition of NF-κB activity reduces LTR-driven transcription. Curcumin has also been reported to influence chromatin remodeling through regulation of histone acetylation status, including modulation of HDAC-dependent pathways. Through these combined effects-destabilization of Tat, suppression of NF-κB activation, and alteration of chromatin accessibility-curcumin promotes a transcriptionally restrictive environment that may limit productive viral replication.

Although curcumin has also been reported in vitro to inhibit HIV-1 integrase and protease activity (Figure 6), these effects are generally considered secondary to its dominant role in modulating host transcriptional and inflammatory pathways [151]. While curcumin’s multi-targeted activity highlights its potential as a host-directed modulator of HIV expression and immune activation, most supporting evidence derives from in vitro studies. Challenges related to bioavailability, dosing, and pharmacokinetics continue to limit its translational application, underscoring the need for further investigation in physiologically relevant models.

### 9.3. Resveratrol

Resveratrol is a naturally occurring polyphenol found in grapes, berries, and red wine and has been extensively studied for its effects on cellular metabolism, aging, and inflammatory regulation. In the context of HIV-1 infection, resveratrol primarily influences viral transcription by modulating host metabolic and epigenetic pathways rather than by directly inhibiting viral enzymes.

A central mechanism underlying resveratrol’s anti-HIV activity involves its ability to increase intracellular nicotinamide adenine dinucleotide (NAD^+^) levels and activate the NAD^+^-dependent deacetylase sirtuin 1 (SIRT1). Activation of SIRT1 leads to deacetylation and functional suppression of the HIV-1 Tat, thereby reducing Tat-mediated transactivation of the HIV-1 LTR (Figure 6). Through this pathway, resveratrol promotes a transcriptionally restrictive environment that limits viral gene expression and may help maintain low-level or suppressed HIV transcription in infected cells under effective ART [152]. In addition, resveratrol has been reported to modulate inflammatory signaling pathways, including suppression of NF-κB activation and downstream pro-inflammatory cytokine production, which may further contribute to a cellular environment that is less permissive for HIV transcription and immune-driven viral reactivation [153]. Beyond its effects on Tat, resveratrol-mediated modulation of cellular metabolism and epigenetic regulation highlights the close relationship between metabolic state and HIV transcriptional control. This link is particularly relevant in the context of viral latency and persistence, where shifts in host metabolic and chromatin-regulatory pathways can influence proviral expression without directly triggering viral reactivation.

Studies have also reported synergistic antiviral effects when resveratrol or its derivatives are combined with nucleoside analogs such as decitabine, resulting in enhanced inhibition of HIV-1 replication in vitro. These findings support further investigation of resveratrol as an adjunctive compound in host-directed HIV therapeutic strategies. However, as with other natural compounds, challenges related to bioavailability, dosing, and pharmacokinetic variability remain significant barriers to clinical translation [153].

### 9.4. Polysaccharide Peptide from Coriolus versicolor

Beyond plant-derived polyphenols, bioactive polysaccharides from medicinal mushrooms have emerged as potent immunomodulatory compounds with relevance to HIV-1 regulation. *Coriolus versicolor* (turkey tail) is a well-characterized medicinal mushroom from which polysaccharide peptide (PSP) has been isolated and extensively studied for its effects on innate and adaptive immune signaling. Unlike small-molecule compounds that directly target viral enzymes, PSP exerts its biological effects primarily through host-directed immune modulation [154,155]. PSP activates innate immune responses through toll-like receptor 4 (TLR4) engagement, leading to downstream activation of antiviral signaling pathways and modulation of cytokine production (Figure 6). In the context of HIV-1 infection, PSP has been shown to significantly restrict viral entry and replication through mechanisms that extend beyond generalized immune activation. Notably, mechanistic studies in THP-1 monocytic cell models have demonstrated that PSP restricts HIV-1 entry by promoting cytoskeletal remodeling via PKR-induced phosphorylation of cofilin-1, a key regulator of actin dynamics required for viral internalization and trafficking. Specifically, PSP has been reported to engage TLR4 and promote downstream upregulation of PKR, which may intersect with non-canonical signaling pathways to induce phosphorylation of cofilin-1 at serine 3. This modification promotes actin polymerization and enhances filamentous actin (F-actin) formation, thereby creating a cortical actin environment that is less permissive to HIV-1 fusion and entry. Through this mechanism, PSP-mediated cytoskeletal remodeling contributes to restriction of early viral internalization events [156]. It disrupts early steps of the HIV-1 life cycle at the level of viral entry, rather than acting solely at the transcriptional or post-integration stages [156].

In HIV-1-infected PBMCs and monocyte-derived models, PSP treatment has been associated with significant reductions in viral replication without detectable cytotoxicity, supporting a host-directed restrictive mechanism. Mechanistically, PSP-mediated TLR4 activation promotes the production of anti-HIV chemokines, including RANTES, MIP-1α/β, and SDF-1α, which competitively inhibit CCR5 and CXCR4 coreceptor usage and thereby limit viral entry. In parallel with its effects on cytoskeletal regulation, this chemokine-driven antiviral environment reinforces a cellular state that is less permissive to HIV-1 infection while preserving immune cell viability and function [157].

Collectively, these findings position mushroom-derived PSP as a distinct class of host-directed immunomodulators with relevance to HIV-1 regulation. PSP, in particular, highlights how modulation of cytoskeletal signaling and innate immune pathways can restrict HIV-1 entry and propagation, providing mechanistic insights into alternative strategies to limit viral spread and persistence. While further studies are required to define its translational potential, PSP provides a compelling example of how natural immunomodulators can inform our understanding of host-controlled HIV expression and immune restriction.

## 10. Therapeutic Implications and Future Directions: Integrating Immune Modulation with LRAs and ARTs

Current HIV cure strategies, including shock-and-kill as well as block-and-lock approaches, seek to either eliminate latent viral reservoirs through controlled reactivation or enforce durable transcriptional silencing while maintaining ART-mediated viral suppression. Central to both strategies is the regulation of HIV expression by host transcriptional, epigenetic, and immune signaling pathways. As such, host-directed immunomodulators, including selected natural compounds, have emerged as potential adjuncts that can influence these regulatory networks without directly targeting viral enzymes [7].

Additional strategies discussed extensively in this review include the use of specific natural compounds that modulate viral restriction, host transcription factors, chromatin accessibility, and immune activation states, which may support latency-reversing or latency-restricting strategies depending on their biological context. Polyphenols such as quercetin and resveratrol have demonstrated context-dependent effects on HIV transcription, functioning as either transcriptional suppressors or enhancers of latency reversal when combined with established LRAs. These findings underscore the importance of cellular environment, dosing, and combinatorial treatment design when considering host-directed approaches to HIV cure strategies [147,148,153].

In parallel, immunomodulatory agents that reinforce innate immune restriction mechanisms may complement block-and-lock-oriented strategies by limiting viral entry, replication, or reactivation without inducing widespread immune activation. Mushroom-derived polysaccharides, including PSP from *Coriolus versicolor*, exemplify this approach by enhancing innate immune signaling and cytoskeletal restriction pathways that reduce cellular permissiveness to HIV infection. Such mechanisms may contribute to sustained viral suppression while preserving immune cell viability and minimizing inflammatory toxicity [156].

Importantly, integration of immunomodulatory compounds with LRAs and ART requires careful consideration of timing, dosing, and immune activation thresholds. Excessive immune stimulation may exacerbate inflammation or tissue damage, particularly within sensitive compartments such as the CNS, whereas insufficient activation may fail to promote clearance. Future studies will therefore need to define optimal combinatorial strategies that balance antiviral efficacy with immune safety, leveraging host-directed modulation to refine curative approaches without compromising long-term immune homeostasis.

## 11. Conclusions

HIV-1 persistence is not governed by a single molecular program but instead emerges from the dynamic interplay between viral factors and host-specific cellular environments. Across canonical and non-canonical reservoirs, differences in chromatin accessibility, transcriptional control, innate immune signaling, and cellular differentiation states collectively determine whether proviral genomes remain transcriptionally silent, intermittently active, or responsive to reactivation cues. This review underscores that viral latency and expression are fundamentally context-dependent processes, shaped by the unique molecular landscapes of CD4+ T cells, myeloid populations, glial cells, and other susceptible cell types. Importantly, mechanisms that restrict viral replication in one cellular compartment may inadvertently stabilize persistence in another, highlighting the limitations of uniform therapeutic approaches. A deeper understanding of host-directed regulatory pathways, ranging from epigenetic modulation and restriction factors to immune-mediated signaling, offers opportunities to refine HIV cure strategies while minimizing immunopathology. Future efforts will require integrated, cell-type-specific frameworks that account for reservoir heterogeneity and immune microenvironmental influences. Such approaches are essential for translating mechanistic insights into effective interventions that durably control or eliminate HIV-1 persistence.

## Data Availability

No new data were created or analysed in this study. Data sharing is not applicable to this article.

## Abbreviations

The following abbreviations are used in this manuscript:

AIDS	Acquired immunodeficiency syndrome
APC	Antigen-presenting cells
APOBEC3G	Apolipoprotein B mRNA editing enzyme catalytic subunit 3G
ART	Antiretroviral therapy
ASC	Apoptosis-associated speck-like protein containing a CARD
BBB	Blood–brain barrier
BETi	Bromodomain and extraterminal domain inhibitor
BST-2	Bone marrow stromal antigen 2
cART	Combination antiretroviral therapy
CCL2	C-C motif chemokine ligand 2
CCL20	C-C motif chemokine ligand 20
CCR5	C-C chemokine receptor type 5
CD4	Cluster of differentiation 4
CD8	Cluster of differentiation 8
CD34	Cluster of differentiation 34
CI	Confidence interval
CNS	Central nervous system
CPSF6	Cleavage and polyadenylation specificity factor 6
CSF-1	Colony-stimulating factor 1
CSF-1R	Colony-stimulating factor 1 receptor
CXCR4	C-X-C chemokine receptor type 4
dNTP	Deoxynucleoside triphosphate
dsRNA	Double-stranded RNA
eIF2α	Eukaryotic translation initiation factor 2-alpha
FISH	Fluorescence in situ hybridization
GALT	Gut-associated lymphoid tissue
Gp120	Glycoprotein 120
H3K36me3	Histone H3 lysine 36 trimethylation
HAND	HIV-associated neurocognitive disorders
HDAC	Histone deacetylase
HDACi	Histone deacetylase inhibitor
HIMEC	Human intestinal microvascular endothelial cell
HIV-1	Human immunodeficiency virus type 1
HMTi	Histone methyltransferase inhibitor
ICAM	Intercellular adhesion molecule 1
IFI16	Interferon gamma-inducible protein 16
IFN	Interferon
IFNAR	Interferon alpha/beta receptor
IFITM3	Interferon-induced transmembrane protein 3
IFITM	Interferon-induced transmembrane protein
IKK	IκB kinase
IL-1β	Interleukin-1 beta
IL-6	Interleukin-6
IL-7	Interleukin-7
IL-15	Interleukin-15
IL-17A	Interleukin-17A
IP-10	Interferon gamma-induced protein 10
IRF3	Interferon regulatory factor 3
ISG	Interferon-stimulated gene
JAK	Janus kinase
KLF2	Krüppel-like factor 2
LEDGF	Lens epithelium-derived growth factor
LFA-1	Lymphocyte function-associated antigen 1
LIMK	LIM kinase 1
LRA	Latency-reversing agent
LTR	Long terminal repeat
MAVS	Mitochondrial antiviral signaling protein
MDA5	Melanoma differentiation-associated protein 5
MHC-I	Major histocompatibility complex class I
MHC-II	Major histocompatibility complex class II
MPS	Mononuclear phagocyte system
MYC	Myelocytomatosis oncogene
NAD^+^	Nicotinamide adenine dinucleotide
Nef	Negative regulatory factor
NFAT	Nuclear factor of activated T cells
NF-κB	Nuclear factor kappa B
NK	Natural killer
NLR	NOD-like receptor
NLRP3	NOD-like receptor family pyrin domain-containing 3
Nuc-1	Nucleosome-1
P-TEFb	Positive transcription elongation factor b
PAMP	Pathogen-associated molecular pattern
PBMC	Peripheral blood mononuclear cell
PCR	Polymerase chain reaction
PKC	Protein kinase C
PKR	Protein kinase R
PRR	Pattern-recognition receptor
PSP	Polysaccharide peptide
RLR	RIG-I-like receptor
RNAP II	RNA polymerase II
ROCK	Rho-associated protein kinase
RTE	Renal tubular epithelial
SAMHD1	Sterile alpha motif and histidine-aspartate domain-containing protein 1
SIRT1	Sirtuin 1
STAT	Signal transducer and activator of transcription
STING	Stimulator of interferon genes
TAR	Transactivation response element
Tat	Transactivator of transcription
TCM	Central memory T cells
TEM	Effector memory T cells
Th	T helper
TLR4	Toll-like receptor 4
TLR	Toll-like receptor
TNF	Tumor necrosis factor
TNF-α	Tumor necrosis factor alpha
UPR	Unfolded protein response
VCAM-1	Vascular cell adhesion molecule 1
VLA-4	Very late antigen 4
